# Urinary Iodine Concentration and Thyroid Hormone Metabolism in Pregnant Women and Neurodevelopment in Their Children: A Longitudinal Canadian Birth Cohort

**DOI:** 10.3390/nu17050830

**Published:** 2025-02-27

**Authors:** Sietske A. Berghuis, Meaghan Hall, John E. Krzeczkowski, Carly V. Goodman, Jonathan Chevrier, Pierre Ayotte, Bruce Lanphear, Christine Till

**Affiliations:** 1 Department of Psychology, York University, Toronto, ON M3J 1P3, Canada; s.a.berghuis@umcg.nl (S.A.B.); mkhall@yorku.ca (M.H.); cgoodman@yorku.ca (C.V.G.); 2 Department of Pediatrics, Beatrix Children’s Hospital, University Medical Center Groningen, University of Groningen, 9713 GZ Groningen, The Netherlands; 3 Department of Health Sciences, Brock University, St. Catharines, ON L2S 3A1, Canada; jkrzeczkowski@brocku.ca; 4 Department of Epidemiology, Biostatistics and Occupational Health, School of Population and Global Health, Faculty of Medicine and Health Sciences, McGill University, Montreal, QC H3A 1G1, Canada; jonathan.chevrier@mcgill.ca; 5 Department of Social and Preventive Medicine, Faculty of Medicine, Université Laval, Québec City, QC G1V 0A6, Canada; pierre.ayotte@inspq.qc.ca; 6 Faculty of Health Sciences, Simon Fraser University, Burnaby, BC V5A 1S6, Canada; bruce_lanphear@sfu.ca

**Keywords:** iodine, creatinine, thyroid, prenatal, child, neurodevelopment, intelligence, externalizing behaviour, internalizing behaviour, autism spectrum disorder

## Abstract

**Background/Objectives**: Iodine is essential for thyroid hormone (TH) synthesis, and THs in pregnant women are critical for fetal brain development. It is unclear whether urinary iodine concentrations (UICs) are associated with thyroid parameters in pregnant women and neurodevelopment in their 3–4-year-old children. **Methods**: In the Canadian Maternal–Infant Research on Environmental Chemicals (MIREC) cohort, we categorized UIC adjusted for urinary creatinine (UIC/Cr) in the first two trimesters as <150, 150–500, or ≥500 µg/g. We used multivariable regression to quantify associations between UIC/Cr and thyroid parameters in maternal plasma (*n* = 1501), including thyroid stimulating hormone (TSH), total T4 (tT4), free T4 (fT4), thyroglobulin (Tg) and Tg antibodies (TgAb), and thyroid peroxidase antibodies (TPOAb). We defined positive thyroid autoantibodies as TgAb ≥ 4.11 or TPOAb ≥ 5.61 IU/mL. We also examined the associations between UIC/Cr with the Wechsler Preschool and Primary Scale of Intelligence (*n* = 503), Behavior Assessment System for Children (*n* = 751), and the Social Responsiveness Scale (*n* = 498). **Results:** Twenty-two percent of women had UIC/Cr < 150 and 17% ≥ 500 µg/g. UIC/Cr was not associated with TSH, tT4, or fT4. After excluding women with positive thyroid autoantibodies, those with UIC/Cr < 150 µg/g had higher tT4 compared to those with 150–500 µg/g. Compared to women with UIC/Cr 150–500 µg/g, those with UIC/Cr < 150 had higher Tg and, those with UIC/Cr ≥ 500 had less frequent positive thyroid autoantibodies. Neurodevelopmental outcomes were not associated with maternal Tg, nor did they differ for maternal UIC/Cr < 150 and ≥500 compared to 150–500 µg/g. **Conclusions:** In this cohort, Tg and tT4 were higher in women with UIC/Cr < 150 µg/g compared to those with UIC/Cr 150–500 µg/g. Urinary iodine in pregnant women was not associated with neurodevelopment in their 3–4-year-old children.

## 1. Introduction

### 1.1. Iodine and Thyroid Hormone Metabolism

Iodine is essential for thyroid hormone (TH) synthesis [1]. Once ingested via food and drinking water or inhalation, iodine is transformed into iodide (I^−^) in the gastrointestinal tract and transported via a sodium/iodine symporter (NIS) from the bloodstream into the cytoplasm of the thyrocyte. Iodide is subsequently transported via membrane enzymes, including thyroid peroxidase (TPO), into the colloid where it binds to thyroglobulin (Tg) to form the THs tetra-iodothyronine (T4; also called thyroxine) and triiodothyronine (T3), which are then proteolyzed in the cytoplasm of the thyrocyte to release T4 and T3 into the bloodstream [2]. In target tissue outside the thyroid gland, the prohormone T4 is converted into the active hormone T3 or into the inactive hormone reverse T3 (rT3) by removal of iodine from the outer or inner ring, respectively [3]. Most of the THs circulating in plasma are protein-bound (including thyroxine-binding globulin and TBG) and do not have biological activity [4]. Only 0.04% of T4 and 0.4% of T3 are not bound to carrier proteins, and this free fraction (fT4 and fT3) is biologically active [4]. Changes in circulating THs influence the secretion of thyroid-stimulating hormone (TSH, also known as thyrotropin) from the pituitary gland via a negative feedback mechanism within the hypothalamic-pituitary-thyroid (HPT) axis [5]. Increasing iodine intake can increase circulating TH concentrations resulting in less secretion of TSH, regulating the synthesis and secretion of Tg [5].

### 1.2. Maternal TH Metabolism and Iodine During Pregnancy

During pregnancy, the demand for thyroid hormone increases as THs are transferred to the fetus, deiodinases in utero-placental tissue activate and inactivate THs, and the peripheral iodothyronine metabolism of maternal thyroid hormones increase [6]. Important physiological changes during pregnancy that influence the maternal thyroid hormone metabolism include increased levels of placental human chorionic gonadotropin beta (β-hCG) and increased levels of maternal estrogen. Placental β-hCG stimulates the maternal thyroid gland, resulting in increased fT4 and T3 [7], which in turn provide negative feedback on the hypothalamus and pituitary, resulting in lower maternal TSH in the first trimester of pregnancy [7]. Maternal estrogen induces a rise in the circulation of TBG, which increases the ability to bind T4, resulting in higher total T4 and lower fT4 [7].

The demand for iodine also increases during pregnancy to meet the need for increased synthesis of THs, transfer of iodine to the fetus, and due to increased renal clearance of iodide [6]. THs are transferred from the mother to the fetus before and after the onset of fetal TH synthesis, which begins around the tenth week of gestation [8]. Disturbances to the maternal thyroid system during this sensitive period of fetal brain development can result in adverse neurodevelopmental outcomes, including a reduction in child intelligence and behavioural problems, with some studies suggesting a greater susceptibility for boys [9,10].

### 1.3. Assessment of Iodine Concentration

Urinary iodine concentration (UIC) is a proxy of recent iodine intake that is subject to high within-person (day-to-day) variability [11]. However, adjusting for creatinine using the urinary iodine to creatinine ratio (UIC/Cr) reduces some of the variability and has been shown to correlate with serum iodine at 8, 20, and 36 weeks gestation and at 6 months postpartum [12]. Concentrations of Tg, the major iodoglycoprotein of the thyroid gland, increase with thyroid hyperplasia found in iodine deficiency [13]. Tg is therefore considered a measure of longer-term iodine status [13] and is negatively associated with iodine excretion [14], which we previously reported in the cohort included in the current study [15].

### 1.4. Iodine Deficiency and Iodine Excess and Maternal TH Metabolism

Iodine deficiency is a risk factor for hypothyroidism in pregnant women, and, in turn, hypothyroidism is a risk factor for diminished intellectual abilities in their offspring [16]. High iodine intake may also affect TH metabolism by increasing the risk for goitre, hypothyroidism, and hyperthyroidism [17]. Urinary iodine concentrations in pregnant women in the first trimester of pregnancy were negatively associated with T4 and free T4 (fT4) and positively associated with maternal TSH in trimester 1 (T1), but results are inconsistent between studies [18,19,20,21,22,23].

### 1.5. Maternal Iodine and Neurodevelopmental Outcomes

Iodine deficiency in pregnancy (defined as UIC/Cr < 150 μg/g) has been linked to poorer behavioural outcomes in children, including a higher risk of attention-deficit hyperactivity disorder (ADHD) in a Dutch cohort, but not in a pooled analysis that included three European cohorts [24]. In a systematic review, low iodine during the first and second trimesters was associated with lower intelligence quotient (IQ) scores and poorer executive functioning in children [25]. None of the 12 studies in the systematic review, however, examined women with excessive iodine intake [25]. Finally, higher maternal serum Tg (<18 weeks of pregnancy) was associated with lower IQ in offspring in early childhood (4.5 and 6 years) but not in later childhood (9 and 13 years) [26].

### 1.6. Aims and Hypotheses

The purpose of this study was to evaluate the cross-sectional association between maternal UIC/Cr and plasma thyroid parameters, as well as the prospective association of maternal iodine status, measured through UIC/Cr and Tg, with child neurodevelopmental outcomes at 3–4 years of age, in a large Canadian pregnancy cohort. In general, the Canadian population is considered iodine-sufficient [27], although a more recent Canadian study found that one-third of nonpregnant women of childbearing age had insufficient iodine intake [28]. We previously characterized this pregnancy cohort as showing a broad range in UIC/Cr (interquartile range, 212–431 ug/g) [15], which provides us with the unique opportunity to explore whether iodine concentrations in the lower and higher ranges are associated with thyroid-related parameters and child neurodevelopment [22]. We hypothesized that both higher and lower markers of maternal iodine status would influence maternal thyroid metabolism and neurodevelopmental outcomes in the offspring.

## 2. Materials and Methods

### 2.1. Cohort Profile

Between 2008 and 2011, the prospective Maternal–Infant Research on Environmental Chemicals (MIREC) study recruited women (*n* = 2001) in the first trimester of pregnancy from prenatal clinics across 10 cities in Canada (Vancouver, Edmonton, Winnipeg, Sudbury, Toronto, Hamilton, Kingston, Ottawa, Montreal, Halifax) [29]. Inclusion criteria for participants in the MIREC study consisted of fluency in English or French, age of 18 years or older, and planning to deliver locally. The MIREC study excluded women with known fetal abnormalities, medical complications, or those using illicit drugs during pregnancy. For the current study, we excluded women with a multiparous pregnancy. For the MIREC-Child Development Plus (MIREC-CD+) study, a subset of women who agreed to be contacted for future research from six of the ten participating sites (Vancouver, Toronto, Hamilton, Kingston, Montreal, Halifax) were invited to participate in in-person neurodevelopmental assessment of their child at three-to-four years of age (*n* = 1459) [30]. Due to resource restrictions, the number of sites was limited, and the sites were selected based on the largest number of children below the age of four. Additionally, mothers from the other four sites were invited to complete the behavioural assessments of their child in a self-administered questionnaire either mailed in paper format or administered via online survey [30]. Both studies were approved by the ethics boards at each recruitment site, Health Canada, and Public Health Agency of Canada (MIREC: REB 2006-027H and MIREC CD+: REB 2012-047H).

### 2.2. Iodine and Creatinine Concentrations During Pregnancy

We collected one spot urine sample in trimester 1 (T1) and another in trimester 2 (T2) [29]. Urine samples were collected in Nalgene^®^ containers (Thermo-Fisher Scientific Inc., Rochester, NY, USA), labelled with a unique identification number, aliquoted into smaller Cryovials, frozen, and shipped to the Toxicology Laboratory at the Institut National de Santé Publique du Québec (INSPQ) for analysis. Urinary iodine concentration (UIC) was measured using inductively coupled plasma mass spectrometry (ICP-MS) as described previously [31]. The limit of detection (LOD) for iodine was 0.3 µmol/L (38 μg/L). Values of UIC below the LOD were replaced by the value of LOD/√2, as recommended by Hornung et al. [32]. The concentration of creatinine in each spot urine sample was measured using a colorimetric end-point assay (Jaffe) on an Indiko instrument (Indiko Plus, ThermoFisher Scientific, Waltham, MA, USA) as described previously [33]. An alkaline sodium picrate solution was used to react with creatinine in urine (UCr) to form a red Janovski complex using Mircogenics DRI^®^ Creatinine-Detect^®^ Test. The absorbance was read at 510 nm on an Indiko chemistry autoanalyzer with a detection limit of 0.069 mmol/L, reporting limit of 0.23 mmol/L, and reproducibility of 2.2% [33].

We calculated the trimester-specific UIC/Cr ratio (μg/g) by dividing UIC (μg/L) by UCr (g/L) to adjust for urine dilution [12]. UCr in mmol/L was converted to mg/dL by dividing UCr by 0.08842 and then converted to g/L by dividing by 100. We used the UIC/Cr measured in the first trimester to investigate the association between UIC/Cr and thyroid parameters. For investigating the associations between UIC/Cr and neurodevelopmental outcomes, we calculated the average UIC/Cr across both trimesters, herein referred to as UIC/Cr_Avg. We categorized maternal UIC/Cr into three groups using pre-defined cut-offs: <150 µg/g, 150–500 µg/g, and >500 µg/g. These categories of UIC/Cr do not necessarily indicate whether the iodine intakes of these pregnant women were insufficient or excessive/high due to large day-to-day variability observed in iodine intake within individuals [11]. The rationale for the grouping is that the cut-off of <150 µg/g is also used in other studies, including UIC/Cr measured in T1 of pregnancy [18,34,35], and the value is close to the cut-off for the first quartile in our cohort (162.8 µg/g). The cut-off >500 µg/g has also been implemented previously in two other studies and approximates the cut-off for our upper quartile (423.7 µg/g) [18,36]. The first study, by Luo et al., defined the cut-off of 489.5 µg/g by using the cut-off for the 97.5 percentile in 285 samples collected between 8 and 12 weeks of pregnancy, and the second study, by Levie et al., also reports on the cut-off of ≥500 µg/g in T1 [18,36]. We also categorized participants into three groups based on the distribution of UIC/Cr in T1 (TH subsample) and UIC/Cr_Avg (neurodevelopment subsample): group 1 consisted of the lowest quartile (Q1), group 2 consisted of the two middle quartiles (Q2 + Q3), and group 3 consisted of the highest quartile (Q4).

### 2.3. Thyroid Parameters During Pregnancy

We collected maternal plasma samples during T1 of pregnancy. Thyroid parameters were quantified as described previously [37]. Plasma tT4 (ng/mL) and fT4 (pg/mL) were quantified using isotope dilution high-performance liquid chromatography–mass spectrometry (ID-HPLC-MS) and gold standard equilibrium dialysis isotope dilution mass spectrometry (ED-ID-MS), respectively, at the Toxicology Laboratory at the INSPQ. For tT4, the LOD was 1.5 ng/mL, and for fT4, it was 1.3 pg/mL. Plasma TSH (μIU/mL), Tg, Tg antibodies (TgAb; IU/mL), and thyroid peroxidase antibodies (TPOAb; IU/mL) were quantified using an Abbott Architect i2000 immunoassay analyzer (Abbott Laboratories, Abbott Park, Chicago, IL, USA) at the Institut Universitaire de Cardiologie et de Pneumologie de Québec (IUCPQ). The LOD for TSH was 0.0025 µIU/mL, and for Tg, it was 0.09 µg/L. For TgAb and TPOAb, the LOD values were 0.07 IU/mL and 0.16 IU/mL, respectively. Values of TSH, Tg, TgAb, and TPOAb below the LOD were replaced by the value of LOD/√2 [32]. Positive thyroid autoantibodies (Thyroid-Ab-pos) were defined by anti-thyroglobulin (anti-Tg) antibody concentrations ≥ 4.11 IU/mL or anti-thyroid peroxidase (anti-TPO) antibody concentrations ≥ 5.61 IU/mL [38,39].

### 2.4. Neurodevelopmental Outcomes

At three-to-four years of age, children completed a neurodevelopmental assessment, and parents filled out questionnaires evaluating their child’s social, emotional, and behavioural functioning [30].

#### 2.4.1. Cognitive Function

The Wechsler Preschool and Primary Scale of Intelligence, Third Edition (WPPSI-III) was used to assess child cognition [40]. The following five subtests were administered: Receptive Vocabulary, Information, Block Design, Object Assembly, and Picture Naming [30]. Three composite scores, full-scale IQ (FSIQ), verbal IQ (VIQ), and performance IQ (PIQ), were calculated using Canadian age-standardized norms. For the composite scores, the internal consistencies are high (Cronbach’s α = 0.90–0.96), and the test–retest reliability coefficients are excellent (intra-class correlation = 0.83–0.90 in children aged 2.5–7.3 years) [41]. VIQ includes the assessment of verbal comprehension, acquired knowledge, and reasoning, while PIQ includes the assessment of non-verbal reasoning, visual–spatial, and visuomotor skills. The WPPSI-III was administered by trained research assistants in participants’ homes, and research staff were blinded to maternal iodine status. A detailed description of the training of the research assistants can be found in the MIREC cohort profile update [30].

#### 2.4.2. Behavioural and Social Functioning

The Behavior Assessment System for Children (2nd Edition; BASC-2) was completed by parents to assess their children’s externalizing and internalizing behaviour problems [42]. Parents completed the BASC-2 either during the scheduled neurodevelopmental visit or by mail or online. The BASC-2 is a 134-item questionnaire with a four-point Likert-type scale, where higher scores represent greater emotional and behavioural difficulties. We used composite T-scores for Externalizing Problems and Internalizing Problems as the main outcome variables of interest. The externalizing scale consists of 22 items assessing hyperactivity and aggression. The internalizing scale consists of 37 items evaluating the presence of anxiety, depression, and somatization. The Social Responsiveness Scale (2nd Edition; SRS-2) Preschool Form was completed by parents during the neurodevelopmental visit to assess the presence and severity of social impairment consistent with autism spectrum disorder (ASD), including questions on interpersonal skills, communication difficulties, and repetitive behaviours [43]. The SRS-2 is a 65-item questionnaire with a four-point Likert scale, where higher scores indicate higher levels of challenge. We used the total T-score, standardized using the questionnaire’s normative data [30].

### 2.5. Covariates

We selected covariates a priori based on prior research on maternal thyroid parameters during pregnancy and neurodevelopmental outcomes [31,37]. For the statistical analyses on the association between maternal UIC/Cr and thyroid parameters during T1, we included the following covariates in the primary model: maternal age (years), race (white/other), pre-pregnancy body mass index (kg/m^2^), parity (0/1/2+), child sex (male/female), and gestational age at collecting plasma samples (weeks). In the secondary model, we included the same covariates and added the following: maternal alcohol intake (yes/no) and second-hand smoke exposure (yes/no) during pregnancy. For the statistical analyses on the association between maternal UIC/Cr_Avg and offspring neurodevelopmental outcomes, we included the following covariates in the primary model: maternal age (years), race (white/other), pre-pregnancy body mass index (kg/m^2^), parity (0/1/ ≥ 2), maternal education level (college diploma or less/university undergraduate or graduate degree), child sex (male/female), and study site. The study site was included in the models for neurodevelopment to control for any potential differences in psychometrists or other exposures that might differ by study site (e.g., community water fluoridation or air pollution). In the secondary model, we added the following covariates: maternal alcohol intake (yes/no) and second-hand smoke exposure (yes/no) during pregnancy, maternal depression (score on the Center for Epidemiological Studies Depression Scale (CES-D 10)), breastfeeding (number of months exclusively breastfeeding), and Home Observation for Measurement of the Environment (HOME) score. Mother–child pairs with missing data on the primary covariates were excluded from the analyses. Because not all participants had data on all covariates, we first studied the associations in our primary model in a larger sample and then further explored the association in our secondary model, including all selected covariates.

### 2.6. Statistical Analyses

We explored the distribution and the descriptive statistics for the demographics, UIC, thyroid parameters, and neurodevelopmental outcomes. Spearman rank correlation tests were used to assess correlations between thyroid parameters. We used Q-Q plots to assess the normality, and we performed natural log transformation for TSH, fT4, and Tg because of a right-skewed distribution.

For the analyses of thyroid parameters, we excluded women who reported using thyroid medication (*n* = 66). For the analyses testing associations between Tg (as an indicator of iodine status) and neurodevelopmental outcomes, we excluded pregnant women with positive thyroid antibodies (Thyroid-Ab-pos) because samples positive for TgAb may result in falsely lowered thyroglobulin concentrations [44]. We used multivariable linear regression to assess the associations between maternal UIC/Cr (continuous; categorized into three groups using pre-defined cut-offs and our quartile-based approach) and maternal plasma TSH, tT4, fT4, and Tg and to assess the associations between maternal iodine status, including UIC/Cr and maternal Tg (as a long-term indicator of iodine status) and offspring neurodevelopmental outcomes. In categorical models, pregnant women with UIC/Cr falling between 150 and 500 µg/g or between the 25th to 75th percentiles served as the reference group. We used UIC/Cr from T1 for thyroid parameter-related analyses because the thyroid parameters were also measured in T1. Regarding the statistical analyses of the association between maternal UIC/Cr during pregnancy and neurodevelopmental outcomes in the offspring, we used UIC/Cr_Avg to have a more robust measure of UIC during pregnancy; however, we also performed trimester-specific analyses. We used multivariable logistic regression to assess whether UIC/Cr is associated with greater odds of Thyroid-Ab-pos.

In addition, we performed sensitivity analyses by running the previously mentioned multivariable regression models excluding women with Thyroid-Ab-pos, which is associated with auto-immune disorder of the thyroid (i.e., Hashimoto’s disease) [45]. Because adjustment for UCr might be less reliable when the urine is too dilute or too concentrated, we reran models, excluding participants with a UCr < 30 mg/dL and >300 mg/dL, for the analyses where there was a statistically significant association [46]. To assess the potential effect modification of child sex, an iodine–sex interaction term was added to the multivariable linear regression models. If the interaction term had a *p*-value < 0.10, we further explored the sex-specific findings by rerunning the model twice, using each sex as the reference group. We ran model diagnostics, including the Breusch–Pagan/Cook–Weisberg test to assess heteroskedasticity, Q-Q plots of the residuals to assess normality of the distribution, Cook’s Distance statistics to assess influential points, and calculation of variance inflation factors to assess multicollinearity between variables. STATA 18 was used for the statistical analyses, and R statistical software version 4.4.2 was used to create the figure in Section 3.7.

With our previously mentioned models, we analyzed the effect of the independent variable UIC/Cr on the dependent variable thyroid hormone parameter. To further explore our data, we assessed whether women meeting the criteria for hypothyroidism had significantly different UIC/Cr compared with women with euthyroid status. Women were classified as primary hypothyroid if their TSH levels were >2.5 μIU/mL and FT4 levels were <11 pg/mL or if they had reported a prior clinical diagnosis of hypothyroidism. Women were classified as subclinical hypothyroid if TSH 2.5–10 μIU/mL and fT4 11–17 pg/mL [47]. For these specific analyses, women who reported using thyroid medication were included in the analyses, and women meeting criteria for subclinical or primary hyperthyroidism were excluded.

## 3. Results

### 3.1. Study Sample Characteristics

**Figure 1:**
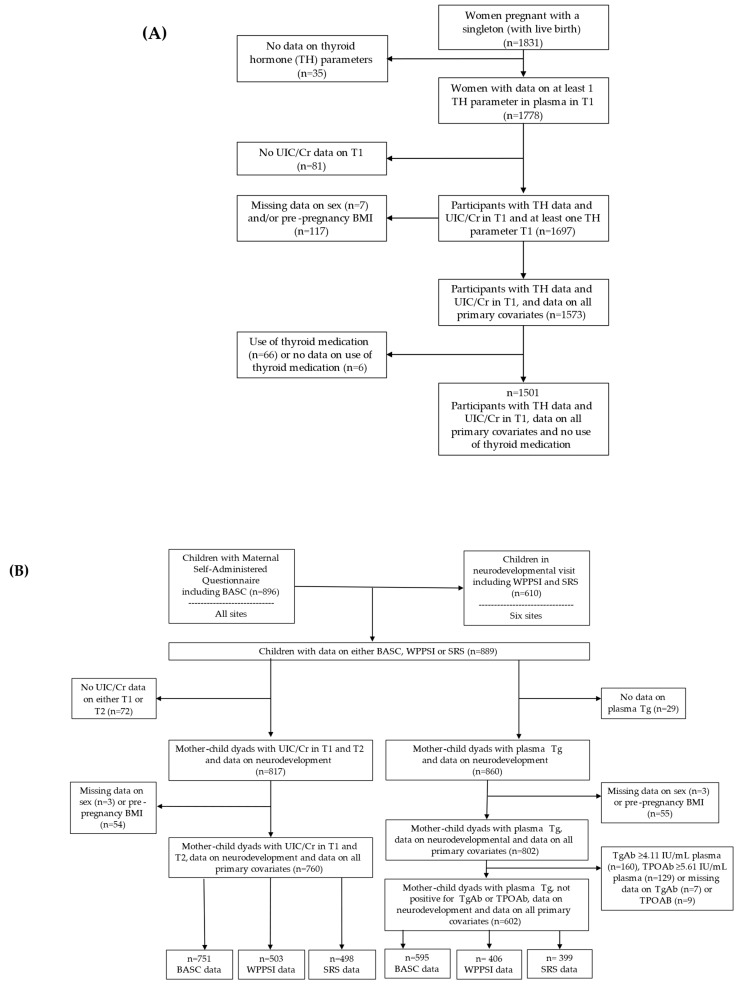
Flowchart of the selection of the participants included in the analyses on (**A**) thyroid and (**B**) neurodevelopmental outcomes.

In total, 1501 pregnant women had data on UIC/Cr in T1, at least one thyroid parameter measured, and data on all primary covariates (Table 1). Of the 1501 mother–child dyads included in the statistical analyses on thyroid parameters, 1314 (88%) pregnant women reported taking a prenatal vitamin, and 800 (53%) had boys. The mean gestational age at blood sampling in T1 was 11.6 weeks (SD: 1.5). Of the 1495 participants invited for an in-person visit for neurodevelopment, a total of 610 families (40.8%) enrolled in the neurodevelopmental visit, of which 600 completed the maternal self-administered questionnaire (MSAQ) including the BASC [30] (Figure 1). In addition, 296 mothers from the sites who were not invited for the onsite neurodevelopmental visit completed the MASQ [30]. In total, 751 had data on behavioural functioning, 503 had data on cognitive functioning, and 498 on social functioning (Figure 1). For the subgroup with neurodevelopmental data and UIC/Cr_Avg, 23% (170 out of the 732) had positive thyroid autoantibodies, 19 (11%) of whom used thyroid medication (Levothyroxine). The mean age at neurodevelopmental testing was 3.4 years (SD: 0.3). The characteristics of the women with data on UIC/Cr and thyroid data (*n* = 1501) and women with data on UIC/Cr and neurodevelopment (*n* = 760) did not significantly differ from the original sample of singleton live births (*n* = 1831), except that the percentage of second-hand smoke during pregnancy was higher in the original sample (5.7%) compared to the neurodevelopment sample (4.5%; Table S1).

### 3.2. Urinary Iodine and Urinary Creatinine Concentration

The UIC and UIC/Cr for those with thyroid parameter data (*n* = 1501) and neurodevelopmental data (*n* = 760) are presented in Table 2. In T1, 10% (153 of 1501) of the samples had a UIC below the LOD, and in T2, 5% (70 of 1395) (Table 2). For the thyroid parameter subsample, 22% had a UIC/Cr < 150 µg/g, and 17% had a UIC/Cr ≥ 500 µg/g in T1. In the neurodevelopmental subsample, 10% of the women had a UIC/Cr_Avg < 150 µg/g, and 18% had a UIC/Cr_Avg ≥ 500 µg/g. Regarding UCr during T1 and T2, 24% (360/1501 and 337/1404, respectively) had a value < 30 mg/dL, and, respectively, 0.6% (*n* = 9) and 0.07% (*n* = 1) had a value > 300 mg/dL.

### 3.3. Thyroid Parameters Concentrations During Pregnancy

In Table 3, we present the plasma concentrations of the thyroid parameters and the proportions of pregnant women with Thyroid-Ab-pos for each of the three categories of UIC/Cr in T1. TSH was below the LOD in three samples (<0.003%), and Tg was below the LOD in 15 samples (<0.01%). For TgAb and TPOAb, 27 (<0.02%) and 178 (12.1%) samples were below the LOD, respectively. TSH was significantly negatively correlated with fT4 and tT4 (*p* < *0*.005), fT4 and tT4 were significantly positively correlated with each other (*p* < 0.005), and Tg was significantly positively correlated with fT4 (*p* < 0.05; Table S2). Plasma concentrations of TgAb and TPOAb were positively correlated with TSH (*p* < 0.01); plasma concentrations of TPOAb were negatively correlated with tT4 and Tg (*p* < 0.05; Table S2).

### 3.4. Neurodevelopmental Outcomes in Offspring at 3–4 Years of Age

In Table 4, we present the neurodevelopmental outcomes stratified by sex. The mean FSIQ was 105 for boys and 109 for girls. The mean composite scores on the SRS-2 and BASC-2 fell within the “average” categories of these norm-referenced questionnaires.

### 3.5. Associations Between Maternal UIC/Cr and Thyroid Parameters

Crude (unadjusted) univariate regression analyses comparing thyroid parameters across the three categories of UIC/Cr show that TSH levels were significantly lower in the UIC/Cr < 150 µg/g group, whereas Tg concentrations were significantly higher in the UIC/Cr < 150 µg/g group. Women with FT4 < 10th percentile (<11 pg/mL) did not have a significantly different mean UIC/Cr and Tg compared to women with FT4 ≥ 10th percentile (UIC/Cr_Avg: 363 versus 349 µg/g; *p* = 0.61; Tg: 15 versus 18 ng/mL; *p* = 0.10). TPOAb levels were lower in the ≥500 µg/g group (Table 3 and Table S3). We used the categorical approach for our analyses because the residuals for the associations with log-transformed TSH, tT4, and log-transformed Tg were not normally distributed when using the continuous UIC/Cr.

The results of the multivariable linear regression analyses for UIC/Cr and thyroid parameters measured in T1 are shown in Table 5. Consistent with our previous work [15], pregnant women with UIC/Cr < 150 μg/g had significantly higher Tg compared to those with UIC/Cr 150–500 μg/g (Table 5). Pregnant women with UIC/Cr < 150 μg/g had 10% lower TSH compared to those with UIC/Cr 150–500 μg/g (B for log-transformed TSH: −0.11; 95% CI: −0.23, −0.00; *p* = 0.05); this association was slightly attenuated and became non-significant after controlling for secondary covariates (Table 5). The sex*UIC/Cr interaction term was significant (*p* = 0.03) for the primary model. Among women pregnant with boys, TSH was 20% lower for those with UIC/Cr < 150 μg/g compared to those with 150–500 μg/g (B for log-transformed TSH = −0.23; 95% CI: −0.38, −0.07). In contrast, no significant differences were found for women pregnant with girls. Multivariable linear and logistic regression analyses showed that participants with UIC/Cr ≥ 500 μg/g had lower odds of having Thyroid-Ab-pos compared to the group of participants with UIC/Cr 150–500 μg/g (Table 5; B −0.46; 95% CI: −0.86, −0.06; *p* = 0.03). The mean UIC/Cr was not significantly different between the 83 women classified as primary hypothyroid (319.7 μg/g) compared with the 1091 women classified as euthyroid (excluding women with subclinical hypothyroidism; 317.4 μg/g; *t*-test *t* = −0.09; *p* = 0.93), and neither between the total of 176 women classified as either primary or subclinical hypothyroid (327.2 μg/g) compared with the women classified as euthyroid (*t*-test *t* = −0.54; *p* = 0.59).

### 3.6. Sensitivity Analyses

#### 3.6.1. UIC/Cr and Thyroid Parameters Excluding Thyroid-Ab-pos

The results of the multivariable linear regression analyses for UIC/Cr and thyroid parameters measured in T1 for the subset without Thyroid-Ab-pos are shown in Table S4. After excluding participants with Thyroid-Ab-pos, pregnant women with UIC/Cr < 150 μg/g had higher tT4 compared to the group of pregnant women with UIC/Cr 150–500 μg/g (Table S4).

#### 3.6.2. UIC/Cr Quartiles and Thyroid Parameters

The results for the quartile-based approach are presented in Table S5. Pregnant women with UIC/Cr in the lowest quartile had significantly lower TSH compared to those with UIC/Cr in the second and third quartiles, and the effect remained significant in the secondary model (Table S5).

#### 3.6.3. UIC/Cr and Thyroid Parameters Excluding Low and High UCr

After excluding participants with UCr < 30 mg/dL and >300 mg/dL, the sample size decreased from 1501 to 1132, and some differences were observed compared to the models that included these participants. Specifically, there was no significant difference in TSH for pregnant women with UIC/Cr < 150 μg/g compared to pregnant women with UIC/Cr 150–500 μg/g (Primary Model: B: −0.05; 95% CI −0.18, 0.07; *p* = 0.37; Secondary Model: B: −0.06; 95% CI −0.19, 0.07; *p* = 0.39). The sex*UIC/Cr interaction term had a *p*-value of 0.09 for the primary model, and rerunning the models with each sex as a reference group did not show significant associations when comparing TSH for those with UIC/Cr < 150 μg/g compared to those with UIC/Cr 150–500 μg/g. We also found that higher tT4 among participants with UIC/Cr < 150 μg/g compared to the group with UIC/Cr 150–500 μg/g was significant in the primary model (B: 3.55; 95% CI 0.18, 6.92; *p* = 0.04; *n* = 849) and was slightly attenuated in the secondary model (B: 3.45; 95% CI: −0.13, 7.03; *p* = 0.06; *n* = 784). Consistent with the results reported above, Tg was significantly higher among those with UIC/Cr < 150 μg/g compared to participants with UIC/Cr 150–500 μg/g, and there was a significantly lower chance of having Thyroid-Ab-pos among those with UIC/Cr ≥ 500 μg/g participants with UIC/Cr 150–500 μg/g.

### 3.7. Associations Between Maternal UIC/Cr_Avg and Offspring Neurodevelopment

**Figure 2:**
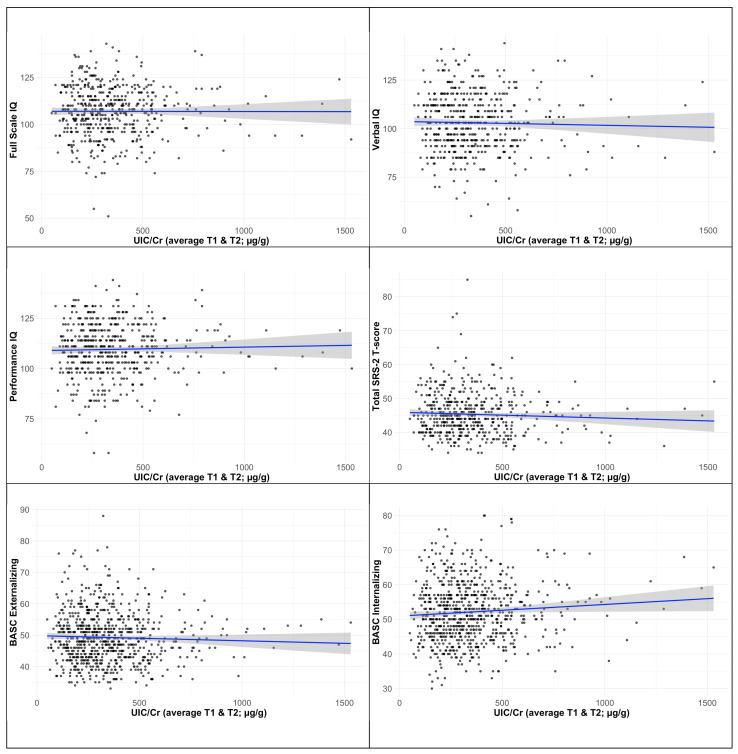
Scatterplots for the associations between UIC/Cr average of the first and second trimester and scores on neurodevelopmental assessments in 3–4-year-old offspring with linear regression lines (blue) and 95% confidence intervals (grey-shaded area). The depicted associations were adjusted for primary covariates. For displaying purposes, two participants with a high UIC/Cr_Avg value (4686 and 6584 μg/g) were excluded when creating the Figures.

Associations between UIC/Cr_Avg and neurodevelopmental outcomes are shown in Figure 2. We used the categorical approach for our analyses on BASC-2 and SRS-2 outcomes because the residuals were not normally distributed or there was heteroscedasticity when using the continuous value of UIC/Cr. For both the continuous values for UIC/Cr and natural log-transformed Tg, as well as for the categorical variable (comparing UIC/Cr < 150 μg/g and UIC/Cr ≥ 500 μg/g with UIC/Cr 150–500 μg/g), no significant associations were found with offspring neurodevelopmental outcomes (Table 6). Null results were also found for the crude models in Table S6, for the quartiles of UIC/Cr (using Q2 + Q3 as the reference group) in Table S7, and for UIC/Cr for both trimesters separately in Table S8 (Table 6 and Tables S6–S8). The only exception was one significant finding in the crude models whereby children whose mothers had high UIC/Cr_Avg had lower SRS-2 scores (i.e., fewer social problems). However, this finding did not remain significant after controlling for covariates.

Regarding sex differences, the natural log-transformed Tg*child sex interaction term had a *p*-value < 0.10 in the model evaluating SRS total T-scores (Table 6). However, the association between log-transformed Tg and SRS total T-score did not reach significance for either sex. The same pattern was observed for T1 but not T2 (Table S8).

## 4. Discussion

### 4.1. Main Findings

We sought to examine the associations between maternal iodine status and thyroid parameters and offspring neurodevelopment in a Canadian prospective pregnancy and birth cohort. Few studies have examined the potential adverse effects of high iodine concentrations in pregnancy. In the current study, both high and low maternal UIC/Cr levels were not associated with TSH, tT4, or fT; after excluding participants positive for thyroid autoantibodies, however, women with UIC/Cr < 150 µg/g had higher tT4 compared to those with 150–500 µg/g. Women with high UIC/Cr (≥500 µg/g) were less likely to have positive thyroid autoantibodies compared to women with UIC/Cr 150–500 µg/g; no associations were found with plasma TSH, fT4, or tT4. No significant associations were observed between low or high maternal UIC/Cr levels, Tg, and any of the child neurodevelopmental outcomes examined, including child IQ, externalizing and internalizing behaviours, and social functioning.

### 4.2. Assessment of Urinary Iodine Concentrations in the MIREC Cohort

Seventeen percent of the women in the current study had a UIC/Cr ≥ 500 µg/g in T1. Other studies reported that fewer than 2% of their participants had UIC/Cr exceeding cut-offs close to or equal to 500 µg/g [18,36]. Consistent with Bath et al., we found that UIC/Cr was higher in T2 compared to T1 [48]. From a mechanistic perspective, this is in line with the hypothesis that during T1, there is an increased demand for iodine and, thus, lower concentrations of iodine excreted in the urine. 

In addition, 22% of women were identified as having UIC/Cr falling in the “low” range using a cut-off of <150 µg/g. As reported previously [15], having a low UIC/Cr was associated with higher Tg compared to UIC/Cr 150–500 μg/g. This is consistent with the study by Bath et al., which found a marginally significantly higher Tg in pregnant women with UIC/Cr < 150 μg/g compared to UIC/Cr ≥ 150 μg/g [34]. A study by Moreno-Reyes et al. reported similar findings, where Tg was marginally significantly higher in those with UIC/Cr < 100 μg/g compared to 100–149, 150–249, and ≥250 μg/g; there was also a significant negative association between UIC/Cr and Tg [20]; the latter was also found by Mullan et al. [49]. In a systematic review and meta-analyses by Dineva et al. on the association between iodine supplementation and thyroid function in pregnant women, most studies reported lower serum Tg concentrations in the iodine supplementation group compared to a placebo group [50], which also suggests a negative association between iodine and Tg concentrations in pregnant women.

Importantly, UIC/Cr derived from one or two spot samples cannot be used to assess the prevalence of inadequate or excessive iodine intake, given that iodine intake can vary considerably from day-to-day. As reported previously, we derived “usual” iodine intake based on two urine samples and adjustment for within-person variation. We then calculated the percentage of our pregnancy cohort with intake below the estimated average requirement (EAR) and above the tolerable upper intake level (UL) using the cut-point method [15]. Using this approach, we found that over 98% of our cohort fell between the EAR of 160 μg/day and UL of 1100 μg/day, indicating sufficient iodine intakes in our pregnancy cohort and likely reflecting the high use of prenatal multivitamins containing iodine [15]. In contrast, a Canadian study of nonpregnant women of childbearing age participating in the Canadian Health Measures Survey (2016–2017) found that iodine intakes were insufficient, with 32% of women having iodine intakes below the EAR [28].

### 4.3. High UIC/Cr and Thyroid Parameters in T1

For pregnant women with UIC/Cr ≥ 500 μg/g, no significant difference in TSH, fT4, and tT4 was found compared to pregnant women with UIC/Cr 150–500 μg/g in T1. To the best of our knowledge, no other studies have compared participants with UIC/Cr ≥ 500 μg/g against groups with lower UIC/Cr. Bath et al. compared participants with UIC/Cr > 250 μg/g to those with 150–249 μg/g and did not find a significant difference in TSH concentrations [34]. Taken together, these findings suggest that even relatively high UIC/Cr does not have a significant effect on maternal TH concentrations.

Participants with UIC/Cr ≥ 500 μg/g were significantly less likely to have Thyroid-Ab-pos in T1 of pregnancy compared to participants with UIC/Cr 150–500 μg/g. In contrast, neither Luo et al. nor Levie et al. found a significant difference in Thyroid-Ab-pos between the group with ‘high’ UIC/Cr (defined as >489 and ≥500 μg/g, respectively) and the reference group (39–489 and 150–249 μg/g, respectively) [18,36]. This inconsistency may be explained by the small proportion of participants (≤1.5%) in past studies with a ‘high’ UIC/Cr. TPOAb and TgAb are shown to be higher (up to 20%) in pregnant women compared to non-pregnant women [51]. Over the timespan of the pregnancy, after initial higher concentrations of thyroid autoantibodies, there is a decrease in concentrations early in pregnancy and an increase in concentrations at the end of pregnancy [51]. The decrease early in pregnancy is suggested to take place to create an immunotolerant environment for the “non-self” (due to paternal origin) fetus [51]. The observed lower thyroid-Ab concentrations in the participants with higher UIC/Cr relative to lower UIC/Cr may suggest that iodine might influence the normal immunologic response during the beginning of pregnancy. Further study is needed to explore whether a high UIC/Cr is associated with Thyroid-Ab at later stages of pregnancy.

### 4.4. Low UIC/Cr and Thyroid Parameters in T1

The participants with low UIC/Cr (<150 μg/g) had lower TSH levels compared to those with UIC/Cr 150–500 μg/g, but this did not remain significant after controlling for alcohol use and second-hand smoke exposure in pregnancy. This is likely due to a reduction in sample size, as the association was not significant when excluding people with missing data on these covariates. In Table S9, we provide an overview of studies on the association between UIC/Cr and thyroid hormone metabolism in T1 (Table S9). We found two other studies also reported a positive association between UIC/Cr and TSH in the first trimester of pregnancy, although they did not report on the association between a categorical variable for UIC/Cr and TSH (Table S9) [18,23]. In contrast, another four studies did not find a significant association between UIC/Cr and TSH in pregnant women during T1 [20,21,22,34]. Our finding showing that TSH levels are lower among the low UIC/Cr group is consistent with the finding that populations with mild-to-moderate iodine deficiency have lower mean TSH than sufficient populations [52]. A suggested mechanism is that the thyroid cells show increased sensitivity to TSH with low iodine content, a phenomenon that has been observed in many clinical studies [53].

Women with UIC/Cr < 150 μg/g did not show different levels of tT4 or fT4 relative to those with UIC/Cr 150–500 μg/g, suggesting that their iodine intake might be adequate enough to not affect the tT4 levels in maternal plasma. However, after excluding pregnant women who were positive for thyroid antibodies, the low UIC/Cr group had *higher* tT4 compared to those with UIC/Cr 150–500 μg/g (Table S4). The presence of thyroid antibodies could be suggestive of an autoimmune condition, resulting in hypothyroidism where tT4 levels drop, consistent with the negative correlation we observed between TPOAb and tT4.

To the best of our knowledge, no other studies reported tT4 on the subgroup of participants with UIC/Cr < 150 μg/g, and the only study reporting on the association between the continuous UIC/Cr and tT4 in T1 of pregnancy reported an inverse association which is in line with our finding [18].

Regarding fT4, we did not see an association with low UIC, even after excluding women who were positive for thyroid antibodies. However, this pattern may differ among women who are being supplemented with iodine. An inverse association was found between fT4 and iodine supplementation (compared to placebo) based on findings in a systematic review and meta-analyses of the effects of iodine supplementation in mildly to moderately iodine-deficient pregnant women [54]. One of the suggested mechanisms for the association between iodine supplementation and fT4 could be due to the Wollf-Chaikoff effect, that excessive amounts of iodide can block the conversion of inorganic iodide to the precursor diiodotyrosine and T4, resulting in *lower* T4, until the iodine concentrations fall below a specific threshold [16].

### 4.5. Iodine Concentrations and Tg During Pregnancy and Neurodevelopmental Outcome

The intrathyroidal iodine reserves and availability of iodine to produce sufficient thyroid hormones are essential for fetal brain development. Regarding our first indirect measure of iodine status, maternal average UIC/Cr, no significant associations were found with offspring neurodevelopmental outcomes, suggesting that intrathyroidal iodine reserves may be more relevant than urinary secretion of iodine. This is consistent with a Dutch study reporting no significant difference in non-verbal IQ and language comprehension in 6-year-old children (*n* = 1525) of women with UIC/Cr < 150 μg/g after adjustment for confounders [55]. In contrast to our findings, Bath et al. reported that 8–9-year-old children of mothers with first-trimester UIC/Cr < 150 μg/g were more likely to score in the lowest quartile for VIQ, reading accuracy and reading comprehension compared to children of women with UIC/Cr ≥ 150 μg/g (women with UIC > 500 μg/L were excluded) in a cohort in the United Kingdom (*n* = 1040) [19]. A possible explanation for this difference in results might be the larger number of pregnant women with a lower UIC/Cr. In the study by Bath et al., 67% (*n* = 646) of the women had a UIC/Cr < 150 μg/g in the first trimester of pregnancy compared to 19% (*n* = 142) of the women in our study. Two additional studies included in a meta-analysis on the effects of iodine supplementation in pregnant women with a mild to moderate iodine deficiency (defined as UIC 50–149 µg/L) both did not find a significant difference between the groups with and without supplementation on scores on the WPPSI-III, global executive composite score from the BRIEF-P, and Bayley Scales of Infant and Toddler Development (Bayley-III) [56,57].

Our second indirect measure of iodine status, plasma Tg, also did not show significant associations with any of the neurodevelopmental outcomes in our study. This is contrary to the findings by Mulder et al. showing that higher Tg was associated with lower IQ in early childhood (age 4.5 and 6 years) in a Spanish and a Dutch cohort [26]. The previously mentioned studies had larger sample sizes (*n* = 749 and 2184 compared to 406), sampling was later in pregnancy compared to our study (13.5 compared to 11.6 weeks), and median UIC/Cr was lower compared to our study (142 and 209 compared to 275 µg/g). Overall, UIC/Cr and plasma Tg during pregnancy were not associated with neurodevelopmental outcomes in our study, which might be due to mechanisms in the thyroid to ensure adequate thyroid hormone production and transfer to the fetus.

### 4.6. Deiodinase Activity and Sodium/Iodine Symporter (NIS)

One of the suggested mechanisms for protecting the fetus from receiving an excessive amount of maternal THs is the activity of deiodinase type 3 (D3). D3 is the predominant iodothyronine deiodinase in the placenta, which can detach an iodine atom from T4 to generate the inactive rT3 and from the active T3 to form 3,5-diiodo-L-thyronine (T2) [6]. Deiodinase type 2 (D2) is also found in the placenta and can detach an iodine atom from the outer ring of T4 to form the active T3, which is suggested to be important for providing adequate T3 concentrations for the developing fetus [6]. In line with this hypothesis, a study in human fetuses found that T4, T3, and deiodinase type 2 (D2) activity were present in the 11th week of gestation [8]. Maternal UIC/Cr was positively associated with fetal cerebral cortex T4 and T3 [8]. Cerebral cortex T4 and T3 peaked during 15–18 weeks of gestation, which is a period during which neuroblastogenesis occurs [8]. In fetuses from women with mild iodine deficiency, compared to fetuses of women with iodine sufficiency, T3 concentrations remain high until the 25th week of gestation (whereas decreasing in the iodine sufficiency group), and D2 activity was higher up to 18 weeks of gestation. This is in line with the hypothesis that increased D2 activity could serve as a compensatory mechanism to maintain adequate fetal T3 during a period of brain development.

Another potential mechanism to prevent the fetus from receiving excessive iodide during a sensitive period of brain development is the regulation of placental NIS. Several factors may be involved in the regulation of NIS expression, including the hCG signal pathway [58]. HCG concentrations are rapidly increasing at the beginning of pregnancy, and hCG has homologies with TSH. As described by Li et al. based on a study in BeWo choriocarcinoma cells (a human cell line used as an in vitro model for the placenta), iodide inhibited NIS expression, NIS mRNA expression, ^125^Iodide uptake, decreased β-hCG mRNA expression, and hCG protein secretion. The addition of hCG to the cells increased NIS mRNA expression, and this was partially inhibited by the addition of iodide [58]. Therefore, they concluded that the expression of placental NIS is modulated by iodide [58]. Regarding thyroid cells, TSH and hCG are suggested to upregulate the NIS expression, whereas iodide is suggested to downregulate both NIS and hCG expression [58]. Down-regulation of the NIS expression in the placenta can result in a reduction in iodide transport to the fetus, which might be a mechanism to protect the fetus from receiving excessive iodide from the mother.

### 4.7. Other Factors Influencing Thyroid Metabolism

Besides suboptimal iodine levels, endocrine-disrupting chemicals can interfere with the thyroid signalling pathway [59]. A first mechanism might be interference with deiodinase activity. For example, higher maternal serum levels of polychlorinated biphenyls have been associated with higher T3 and lower rT3 in cord blood, which might be due to reduced activity of deiodinase type 3 during pregnancy [60]. Because everyone is exposed to a mixture of chemicals, it is important to understand whether chemicals can interfere with deiodinase activity and other homeostatic mechanisms that prevent the fetus from receiving excess or deficient iodide concentrations, which is essential for TH concentrations and the development of the fetal thyroid system.

A second mechanism for the effects of chemicals on iodine and thyroid metabolism might be the inhibition of NIS gene expression. For example, fluoride can inhibit both NIS gene expression and sodium potassium-activated adenosine 5′-triphosphatase pump (Na+, K+-ATPase) activity [61]. The Na+, K+-ATPase is important for iodine transport and the functionality of NIS [61]. Waugh provided an overview of studies supporting evidence that dietary iodine absorption and incorporation can be reduced by fluoride [61]. The offspring of pregnant women with iodine deficiency appear to be at an increased risk for the neurotoxic effects of chemicals. In the current cohort, maternal urinary fluoride concentrations were found to be associated with lower FSIQ in boys, and this association was stronger among boys whose mothers had low urinary iodine concentrations (which was defined as UIC/Cr < 200 μg/g) in pregnancy compared to boys whose mothers had adequate iodine concentrations (defined as ≥200 to 600 μg/g) in pregnancy [31]. Perchlorate, thiocyanate, and nitrate are sodium/iodide symporter (NIS) inhibitors that block iodide uptake into the thyroid, thus affecting thyroid function [62]. Per- and polyfluoroalkyl substances (PFAS), a class of persistent chemicals, can also act as NIS inhibitors [63]. Webster et al. found evidence of PFAS-associated thyroid disruption in adults with both high TPOAb and lower urinary iodine concentrations [64]. These studies suggest that iodine deficiency enhances the neurotoxicity of prevalent toxic chemicals, such as fluoride, PFAS, and perchlorate. If so, understanding the impact of iodine deficiency on neurodevelopment will require simultaneously examining how prevalent thyroid disruptors modify its associations; studying iodine deficiency alone may not be sufficient.

### 4.8. Strengths and Limitations

This study had strengths and limitations. We have a relatively large sample size, a broad range of UIC/Cr concentrations, and a relatively large group with UIC/Cr ≥ 500 µg/g, enabling us to assess whether a higher urinary iodine concentration would be associated with neurodevelopmental outcomes in preschool-aged children. Another important strength is that we measured Tg, a longer-term estimate of iodine status, and could adjust our models for a number s of potential confounding factors. 

Limitations included only collecting one or two urine samples for UIC/Cr measurements, and not having information on when the pregnant women ingested their prenatal vitamins (most of which contain iodine in Canada) or if the women had ingested iodine-rich food prior to urine sampling. One or two urine samples are not enough to fully characterise the iodine status of an individual. The time of the most recent iodine supplement intake prior to sampling is considered an important factor for urinary iodine concentration [65]. We can, therefore, not exclude the chance that a high UIC in pregnant women was due to recent intake of iodine supplements or consuming iodine-rich food. An additional complicating factor of considering only present iodine level is that even small increases in iodine intake, for example, through prenatal supplementation, may change the pattern of thyroid disease among previously iodine-deficient individuals [66]. To provide a more robust approach, we used the average of the UIC/Cr measured in two trimesters as well as Tg (as a marker of longer-term iodine status) for the analyses on the association between UIC/Cr and child neurodevelopmental outcome. We also reported the results by categories of UIC/Cr, which limits the effect of individual data points with high or low UIC/Cr values. Future studies should consider the use of iodine supplements and time between the most recent iodine supplement prior to urine sampling and can consider collecting 24 h urine samples to be able to adjust for UIC fluctuations throughout the day. 

The second limitation is that one-quarter of the samples had creatinine concentrations below 30 mg/dL. Previous studies have suggested that this might limit the reliability of the method to adjust for creatinine, and the World Health Organization (WHO) recommends taking another urine sample for the measurement of creatinine [46,67]. We therefore performed sensitivity analyses excluding participants with urinary creatinine concentrations < 30 mg/dL and >300 mg/dL to account for this. Most, but not all, associations remained significant after excluding participants with urinary creatinine concentrations < 30 mg/dL and >300 mg/dL, suggesting, i.e., that the observed associations between UIC/Cr and high tT4 were also present when excluding participants with more diluted urine.

The third limitation is that we did not measure iodine concentration in serum. Li et al. concluded that for the diagnosis of iodine excess (defined as unadjusted UIC > 500 µg/L), serum iodine can be a better diagnostic indicator compared to UIC/Cr, though both are considered good diagnostic indicators (area under the ROC curve 0.82 and 0.75, respectively). For the diagnosis of iodine deficiency (defined by the WHO as unadjusted UIC < 150 g/L), UIC/Cr is considered a better diagnostic indicator compared to serum iodine. Nevertheless, we believe that UIC/Cr is considered a reliable diagnostic tool, as studies showed that UIC/Cr was significantly correlated with serum iodine [12], and we found that UIC/Cr was associated with plasma Tg.

The fourth limitation is that we do not have data on the UIC/Cr before pregnancy. On a population level, the introduction of iodine supplementation programs in iodine-deficient populations has been suggested to induce iodine-induced hyperthyroidism in the first years after implementation [68,69]. Sohn et al. also described that an acute iodine load may result in hypo- or hyperthyroidism in certain vulnerable persons [70]. Therefore, it seems important to consider the UIC/Cr before pregnancy as a factor in evaluating the effects of iodine on the TH metabolism during pregnancy.

The fifth limitation is the possibility of selection bias. The cohort consists of a relatively large number of pregnant women with higher education levels, and women who agreed to participate in a study on the effects of environmental chemical exposure may be more conscious of their daily life choices related to exposure to chemicals and a healthy diet during pregnancy. The characteristics of women with data on neurodevelopmental outcomes in their offspring are almost similar compared to the overall MIREC cohort. In our cohort, 88% of the pregnant women used prenatal vitamins, and as reported previously, prenatal vitamin use was found to be associated with UIC, which might be because most prenatal vitamins in Canada include iodine [15]. This might have resulted in missing a group of pregnant women with possibly higher risks of impact on either low or high iodine intake and limits the generalizability of the results to the broader Canadian population.

## 5. Conclusions

In this longitudinal Canadian birth cohort study, 22% of pregnant women had a UIC/Cr < 150 µg/g and 17% ≥ 500 µg/g in the first trimester of pregnancy. After excluding participants positive for thyroid autoantibodies, we found that women with UIC/Cr in the lower range (<150 µg/g) had higher plasma tT4 compared to those with UIC/Cr between 150 and 500 µg/g. Women with UIC/Cr in the higher range (≥500 µg/g) were less likely to have positive thyroid autoantibodies compared to women with UIC/Cr 150–500 µg/g, and no associations were found with plasma TSH, fT4, or tT4. These findings suggest that even a relatively high maternal urinary iodine concentration during pregnancy is not associated with changes in maternal THs, which might be due to compensatory mechanisms across the maternal, placental, and fetal thyroid hormone systems to maintain adequate TH supply to the fetus. Maternal UIC/Cr levels during pregnancy were not significantly associated with neurodevelopment in 3–4-year-old offspring.

## Figures and Tables

**Table 1:** Characteristics of the study group.

	Thyroid Hormone Data and UIC/Cr T1 ^a^ (*n* = 1501)	Neurodevelopmental Data and UIC/Cr_Avg ^b^ (*n* = 760)
**Maternal characteristics**		
Maternal age at pregnancy (years; mean ± SD)	32.3 ± 5.0	32.8 ± 4.7
Race (*n*; %)		
White	1287 (86%)	690 (91%)
Other	214 (14%)	70 (9%)
Level of education of mother at pregnancy (*n*; %)		
College diploma or less	551 (37%)	241 (32%)
University undergraduate or graduate degree	950 (63%)	519 (68%)
Pre-pregnancy BMI (kg/m^2^; mean; SD)	24.8 ± 5.4	25.0 ± 5.6
Parity (*n*; %)		
0	661 (44%)	339 (45%)
1	611 (41%)	310 (41%)
≥2	229 (15%)	111 (15%)
CESD-10 mother (*n* = 731; mean; range)	∙	5.6 (0–23)
Alcohol use during pregnancy ^c^ (*n*; %)	250 (18%)	134 (18%)
Second-hand smoke during pregnancy ^d^ (*n*; %)	83 (5.5%)	34 (4.5%)
Use of thyroid medication ^e^ (*n*; %)	∙	32 (4.2%)
Maternal thyroid auto antibodies in T1		
TgAb-positive ^f^ (≥4.11 IU/mL plasma; *n*; %)	279 (19%)	144 (19%)
TPOAb-positive ^g^ (≥5.61 IU/mL plasma; *n*; %)	192 (13%)	120 (17%)
Use of prenatal vitamins ^h^	1314 (88%)	660 (87%)
**Child characteristics**		
Child male sex (*n*; %)	800 (53%)	364 (48%)
Gestational age in weeks at birth (mean ± SD)	∙	39.5 ± 1.6
Number of months exclusively breastfeeding (*n* = 754; mean ± SD)	∙	5.0 ± 3.2
HOME total score (*n* = 489; mean ± SD)	∙	47.5 ± 4.3
Child age at WPPSI and SRS in years (*n* = 505; mean ± SD)	∙	3.4 ± 0.3

Abbreviations: UIC = urinary iodine concentration; Cr = creatinine; T1 = trimester 1; SD = standard deviation; BMI = body mass index; CESD = Center for Epidemiological Studies Depression Scale; TgAb = thyroglobulin antibodies; TPOAb = thyroid peroxidase antibodies; HOME = Home Observation for Measurement of the Environment; WPPSI = Wechsler Preschool and Primary Scale of Intelligence; SRS = Social Responsiveness Scale; ^a^ Data presented for subgroup with thyroid parameters is including pregnant women with UIC/Cr in the first trimester (T1) without using thyroid medication; ^b^ Data presented for subgroup with neurodevelopment is including women with UIC/Cr in T1 and T2 and pregnant women using thyroid medication; ^c^ women who reported ever drinking alcohol during pregnancy; 127 and 19 missing data; ^d^ 2 missing for thyroid parameter subgroup; ^e^ 3 with missing data; ^f^ 20 and 21 missing; ^g^ 27 and 22 missing; ^h^ 1 with missing data.

**Table 2:** Iodine and creatinine concentrations in spot urine samples from pregnant women.

	Thyroid Parameter Data and UIC/Cr T1 ^a^ (*n* = 1501)	Neurodevelopmental Data and UIC/Cr T1 and T2 ^b^ (*n* = 760)
UIC/Cr Average T1 and T2 (μg/g; median; IQR)	∙	310.5 (216.2–446.6) ^c^
<150 μg/g	∙	77 (10%)
150–500 μg/g	∙	549 (72%)
≥500 μg/g	∙	134 (18%)
UIC/Cr T1 (μg/g; median; IQR)	266.5 (162.8–423.7) ^c,d^	275.0 (169.6–456.8) ^c,d^
<150 μg/g	328 (22%)	142 (19%)
150–500 μg/g	925 (62%)	479 (63%)
≥500 μg/g	248 (17%)	139 (18%)
UIC/Cr T2 (μg/g; median; IQR)	∙	317.6 (200.7–463.5) ^c,e^
<150 μg/g	∙	102 (13%)
150–500 μg/g	∙	497 (65%)
≥500 μg/g	∙	161 (21%)
Tg in plasma (ng/mL; median; IQR)	13.9 (8.7–21.5) ^f^	14.2 (8.4–21.7) ^g^
Urinary creatinine T1 (mg/dL; mean ± SD)	76.9 ± 59.7	75.5 ± 57.7
Urinary creatinine T2 (mg/dL; mean ± SD)	∙	69.9 ± 52.2
Unadjusted urinary iodine T1 (μg/L; median; IQR)	151.9 (75.9–278.5) ^c,d^	158.2 (77.2–303.8) ^c,d^
<150 μg/L (*n*; %; “Insufficient”)	717 (48%)	350 (46%)
150–249 μg/L (*n*; %; “Adequate”)	341 (23%)	169 (22%)
250–499 μg/L (*n*; %; “Above requirements”)	326 (22%)	172 (23%)
≥500 μg/L (*n*; %; “Excessive”)	117 (8%)	69 (9%)
Unadjusted urinary iodine T2 (μg/L; median; IQR)	∙	164.6 (88.6–303.8) ^c,e^
<150 μg/L (*n*; %; “Insufficient”)	∙	336 (44%)
150–249 μg/L (*n*; %; “Adequate”)	∙	173 (23%)
250–499 μg/L (*n*; %; “Above requirements”)	∙	184 (24%)
≥500 μg/L (*n*; %; “Excessive”)	∙	67 (9%)

Abbreviations: UIC = urinary iodine concentration; Cr = creatinine; T1 = trimester 1; T2 = trimester 2; IQR = interquartile range; SD = standard deviation; ^a^ Data presented for subgroup with thyroid parameters is including pregnant women with UIC/Cr in trimester 1 without using thyroid medication; ^b^ Data presented for subgroup with neurodevelopment is including women with UIC/Cr in both trimesters and pregnant women using thyroid medication; ^c^ values below the limit of detection (LOD) were replaced by LOD/√2; ^d^ *n* = 153 and *n* = 70 with UIC < LOD; ^e^ *n* = 38 with UIC < LOD; ^f^ 16 missing; ^g^ 18 missing.

**Table 3:** Thyroid parameter concentrations and antibodies in maternal plasma during first trimester in urinary iodine/urinary creatinine ratio in 3 categories for mother–child dyads with thyroid outcome measures (excluding women taking thyroid medication).

UIC/Cr Trimester 1 (μg/g)	TSH (μIU/mL) ^a^ (*n* = 1426)		tT4 (ng/mL) (*n* = 1499)		fT4 (pg/mL) (*n* = 1478)		Tg (ng/mL) ^b^ (*n* = 1485)		TgAb (IU/mL) ^c^ (*n* = 1481)		TPOAb (IU/mL) ^d^ (*n* = 1474)		TgAb ≥ 4.11 IU/mL; *n* (% of Participants in Category) (*n* = 1481)	TPOAb ≥ 5.61 IU/mL; *n* (% of Participants in Category) (*n* = 1474)
<150	308	1.26 ± 1.07 ^e^	328	107.1 ± 19.2	322	13.4 ± 2.4	324	20.2 ± 18.5 ^e^	322	12.3 ± 57.1	320	25.0 ± 98.5	61 (19%)	47 (15%)
150–500	885	1.37 ± 0.98 ^e^	924	104.8 ± 20.8	911	13.5 ± 2.7	915	17.0 ± 15.7 ^e^	913	10.9 ± 53.9	908	21.2 ± 83.8 ^f^	184 (20%) ^f^	125 (14%) ^f^
≥500	233	1.38 ± 0.82	247	106.2 ± 20.5	245	13.3 ± 2.5	246	16.3 ± 11.9	246	7.7 ± 39.9	246	19.3 ± 105.7 ^f^	34 (14%) ^f^	20 (8%) ^f^

Abbreviations: UIC = urinary iodine concentration; Cr = creatinine; T1 = trimester 1; TSH = thyroid stimulating hormone; T4 = thyroxine; tT4 = total T4; fT4 = free T4; Tg = thyroglobulin; TgAb = Tg antibodies; TPO = thyroid peroxidase; TPOAb = TPO antibodies; data are reported as mean ± SD or *n* (%); values below the limit of detection (LOD) are replaced by LOD/√2; ^a^ *n* = 3 < LOD; ^b^ *n* = 15 < LOD; ^c^ *n* = 27 < LOD; ^d^ *n* = 178 < LOD; ^e^ comparison between <150 μg/g versus 150–500 μg/g using log-transformed outcomes (TSH, Tg) is statistically significant, *p* < 0.05; ^f^ comparison between ≥500 μg/g versus 150–500 μg/g using log-transformed TPOAb is statistically significant, *p* < 0.05.

**Table 4:** Neurodevelopmental outcomes in mother–child dyads with data on UIC/Cr in both trimesters ^a^.

Neurodevelopmental Outcomes	*n*	Boys	Girls
WPPSI Full Scale IQ (mean ± SD)	503	104.5 ± 14.2	109.2 ± 12.1
WPPSI Verbal IQ (mean ± SD)	500	107.1 ± 13.8	111.9 ± 12.1
WPPSI Performance IQ (mean ± SD)	498	101.4 ± 15.1	104.4 ± 14.0
SRS-2 Total T-score (mean ± SD)	498	46.6 ± 6.7	44.3 ± 5.6
BASC-2 T-score Composite score Externalizing Problems (mean ± SD)	751	50.7 ± 8.2	48.0 ± 7.7
BASC-2 T-score Composite score Internalizing Problems (mean ± SD)	742	52.0 ± 8.4	52.2 ± 8.7

Abbreviations: UIC = urinary iodine concentration; Cr = creatinine; WPPSI = Wechsler Preschool and Primary Scale of Intelligence; IQ = intelligence quotient; SD = standard deviation; SRS = Social Responsiveness Scale; BASC = Behavior Assessment System for Children; ^a^ data presented include pregnant woman using thyroid medication.

**Table 5:** Multivariable linear and logistic regression analyses for maternal urinary iodine concentration divided by urinary creatinine concentration and thyroid hormone metabolism parameters during trimester 1 of pregnancy (excluding women using thyroid medication).

Thyroid Parameter	UIC/Cr T1 Categories	Primary Model ^a^				Secondary Model ^b^			
		*n*	B	95% CI	*p*	*n*	B	95% CI	*p*
Log TSH (µIU/mL)	<150 versus 150–500 µg/g	1426	−0.11	−0.23, −0.00	**0.05**	1305	−0.10	−0.22, 0.02	0.11
	≥500 versus 150–500 µg/g		−0.02	−0.14, 0.10	0.75		−0.01	−0.14, 0.12	0.91
Log fT4 (pg/mL)	<150 versus 150–500 µg/g	1478	−0.00	−0.03, 0.02	0.67	1354	−0.01	−0.03, 0.02	0.48
	≥500 versus 150–500 µg/g		−0.02	−0.05, 0.01	0.12		−0.02	−0.04, 0.01	0.20
tT4 (ng/mL)	<150 versus 150–500 µg/g	1499	1.74	−0.81, 4.28	0.18	1372	2.35	−0.34, 5.04	0.09
	≥500 versus 150–500 µg/g		1.28	−1.54, 4.11	0.37		1.77	−1.13, 4.68	0.23
Log Tg (ng/mL)	<150 versus 150–500 µg/g	1485	0.19	0.05, 0.33	**0.01**	1358	0.18	0.03, 0.33	**0.02**
	≥500 versus 150–500 µg/g		0.03	−0.13, 0.18	0.74		0.03	−0.13, 0.19	0.72
**Logistic Regression**									
TgAb ≥ 4.11 IU/mL (versus <4.11)	<150 versus 150–500 µg/g	1481	−0.06	−0.39, 0.27	0.72	1354	−0.06	−0.41, 0.29	0.75
	≥500 versus 150–500 µg/g		−0.46	−0.86, −0.06	**0.03**		−0.44	−0.86, −0.03	**0.04**
TPOAb ≥ 5.61 IU/mL (versus <5.61)	<150 versus 150–500 µg/g	1474	0.11	−0.25, 0.48	0.54	1348	0.05	−0.35, 0.44	0.82
	≥500 versus 150–500 µg/g		−0.60	−1.10, −0.10	**0.02**		−0.62	−1.13, −0.11	**0.02**

^a^ Primary Model: maternal age, race, pre-pregnancy body mass index, parity, child sex and age at sampling; ^b^ Secondary Model: maternal age, race, maternal pre-pregnancy body mass index, parity, child sex, age at sampling and maternal alcohol use during pregnancy, second-hand smoke during pregnancy. Bold indicates significant findings.

**Table 6:** Multivariable linear regression analyses for maternal urinary iodine concentration divided by urinary creatinine concentration during pregnancy (average T1 and T2) and neurodevelopmental outcomes in offspring (including women using thyroid medication).

		Primary Model ^a^				Secondary Model ^b^				Interaction Term Sex ^c^
		*n*	B	95% CI	*p*	*n*	B	95% CI	*p*	*p*
WPPSI-Full Scale IQ	UIC/Cr Average T1 and T2 (μg/g × 0.01)	503	−0.03	−0.36, 0.30	0.86	455	0.01	−0.32, 0.33	0.97	0.27
	<150 versus 150–500 µg/g		−0.37	−3.94, 3.21	0.84		−1.45	−5.05, 2.14	0.43	
	≥500 versus 150–500 µg/g		−0.56	−3.60, 2.47	0.72		−0.85	−3.93, 2.24	0.59	
	Log Tg ^d^	406	0.17	−1.72, 2.05	0.86	361	0.56	−1.35, 2.46	0.57	0.88
WPPSI-Verbal IQ	UIC/Cr Average T1 and T2 (μg/g × 0.01)	500	−0.05	−0.37, 0.27	0.74	452	−0.03	−0.35, 0.29	0.85	0.32
	<150 versus 150–500 µg/g		0.23	−3.29, 3.75	0.90		−0.63	−4.21, 2.94	0.73	
	≥500 versus 150–500 µg/g		0.10	−2.86, 3.07	0.95		−0.38	−3.41, 2.66	0.81	
	Log Tg ^d^	404	−0.19	−1.99, 1.61	0.84	359	0.19	−1.68, 2.06	0.84	0.89
WPPSI-Performance IQ	UIC/Cr Average T1 and T2 (μg/g × 0.01)	498	0.02	−0.34, 0.38	0.90	451	0.06	−0.30, 0.43	0.73	0.31
	<150 versus 150–500 µg/g		−0.75	−4.69, 3.18	0.71		−1.69	−5.72, 2.34	0.41	
	≥500 versus 150–500 µg/g		−1.10	−4.46, 2.26	0.52		−0.99	−4.47, 2.48	0.57	
	Log Tg ^d^	401	0.38	−1.69, 2.44	0.72	357	0.86	−1.27, 2.98	0.43	0.89
SRS-Total T-Score	UIC/Cr Average T1 and T2 (μg/g × 0.01)	498				451				
	<150 versus 150–500 µg/g		−0.53	−2.23, 1.18	0.54		−0.13	−1.85, 1.59	0.88	
	≥500 versus 150–500 µg/g		−1.26	−2.70, 0.18	0.09		−1.06	−2.53, 0.41	0.16	
	Log Tg ^d^	399	−0.12	−1.02, 0.78	0.80	356	−0.14	−1.07, 0.78	0.77	0.04
	boys as reference	399	−1.07	−2.33, 0.19	0.10	356	−1.20	−2.48, 0.09	0.07	
	girls as reference	399	0.83	−0.43, 2.10	0.20	356	0.96	−0.35, 2.27	0.15	
BASC-T-score Composite score Externalizing Problems	UIC/Cr Average T1 and T2 (μg/g × 0.01)	751				455				
	<150 versus 150–500 µg/g		0.57	−1.35, 2.50	0.56		0.62	−1.62, 2.86	0.59	
	≥500 versus 150–500 µg/g		−0.97	−2.51, 0.58	0.22		−1.07	−2.98, 0.85	0.27	
	Log Tg ^d^	595	−0.13	−1.06, 0.79	0.77	361	0.12	−1.09, 1.33	0.85	0.74
BASC-T-score Composite score Internalizing Problems	UIC/Cr Average T1 and T2 (μg/g × 0.01)	744				453				
	<150 versus 150–500 µg/g		0.55	−1.52, 2.62	0.60		1.45	−0.83, 3.74	0.21	
	≥500 versus 150–500 µg/g		0.87	−0.79, 2.53	0.30		0.94	−1.02, 2.91	0.35	
	Log Tg ^d^	588	−0.83	−1.81, 0.15	0.10	359	−0.02	−1.24, 1.21	0.98	0.93

^a^ Primary Model: maternal age, race, pre-pregnancy body mass index, parity, maternal education level, child sex, and study site; ^b^ Secondary Model: maternal age, race, maternal pre-pregnancy body mass index, parity, maternal education level, child sex, maternal alcohol use during pregnancy, second-hand smoke during pregnancy, depression CES-D 10 score mother, breastfeeding, study site and HOME score; ^c^ in primary model; ^d^ excluding women with TgAb ≥ 4.11 IU/mL or TPOAb > 5.61 IU/mL, including women using thyroid medication.

## Data Availability

Restrictions apply to the availability of these data. Source data were and can be accessed through an application and review process as required by MIREC’s Biobank governance, as described at: https://www.mirec-canada.ca/en/research/.

## Supplementary Materials

The following supporting information can be downloaded at: https://www.mdpi.com/article/10.3390/nu17050830/s1, Table S1: Characteristics of the full sample (*n* = 1831) and the sample with data on UIC/Cr, thyroid hormone data or neurodevelopmental outcome, and complete data on primary covariates. Table S2: Spearman rank correlation test between thyroid parameters during the first trimester of pregnancy within the MIREC cohort. Table S3: Univariate linear and logistic regression analyses for maternal urinary iodine concentration divided by urinary creatinine concentration and thyroid hormone metabolism parameters during trimester 1 of pregnancy (excluding women using thyroid medication). Table S4: Multivariable linear regression analyses for maternal urinary iodine concentration divided by urinary creatinine concentration and thyroid hormone metabolism during trimester 1 of pregnancy (excluding women using thyroid medication and with positive thyroid autoantibodies). Table S5: Multivariable linear regression analyses for maternal urinary iodine concentration divided by urinary creatinine concentration in quartiles and thyroid hormone metabolism during trimester 1 of pregnancy (excluding women using thyroid medication). Table S6: Univariate linear regression analyses for maternal urinary iodine concentration divided by urinary creatinine concentration during pregnancy (average T1 and T2) and neurodevelopmental outcomes in offspring (including woman using thyroid medication). Table S7: Multivariable linear regression analyses for maternal urinary iodine concentration divided by urinary creatinine concentration during pregnancy in quartiles and neurodevelopmental outcomes in offspring. Table S8: Multivariable linear regression analyses for maternal urinary iodine concentration divided by urinary creatinine concentration during pregnancy in the first and second trimester and neurodevelopmental outcomes in offspring (*n* T1 = 796 T1 and *n* T2 = 783; including women using thyroid medication). Table S9: Overview of studies on UIC/Cr and thyroid hormone parameters during the first trimester of pregnancy. References [71,72] are cited in Supplementary Materials.

## Abbreviations

The following abbreviations are used in this manuscript:

ASD	autism spectrum disorder
BASC-2	Behavior Assessment System for Children, Second Edition
β-hCG	human chorionic gonadotropin beta
BMI	body mass index
CES-D 10	Center for Epidemiological Studies Depression Scale
D2	deiodinase type 2
D3	deiodinase type 3
EAR	estimated average requirement
hCG	human chorionic gonadotropin
HOME score	Home Observation for Measurement of the Environment score
HPT axis	hypothalamic-pituitary-thyroid axis
I^−^	iodide
ICP-MS	inductively coupled plasma mass spectrometry
INSPQ	Institut National de Santé Publique du Québec
IQ	intelligence quotient
IUCPQ	Institut Universitaire de Cardiologie et de Pneumologie de Québec
LOD	limit of detection
FSIQ	full-scale IQ
fT3	free T3
fT4	free T4
MSAQ	maternal self-administered questionnaire
MIREC study	Maternal–Infant Research on Environmental Chemicals study
MIREC-CD+ study	MIREC-Child Development Plus study
Na+, K+-ATPase	sodium potassium-activated adenosine 5′-triphosphatase pump
NIS	sodium/iodine symporter
PFAS	per- and polyfluoroalkyl substances
PIQ	performance IQ
rT3	reverse T3
SRS-2	Social Responsiveness Scale, Second Edition
T1	trimester 1
T2	trimester 2
T3	triiodothyronine
T4	tetra-iodothyronine thyroxine
tT4	total T4
TBG	thyroxine-binding globulin
Tg	thyroglobulin
TgAb	Tg antibodies
TH	Thyroid hormone
Thyroid-Ab-pos	positive thyroid antibodies
TPO	thyroid peroxidase
TPOAb	TPO antibodies
TSH	thyroid-stimulating hormone
UCr	urinary creatine concentration
UIC	urinary iodine concentration
UIC/Cr	urinary iodine concentration divided by urinary creatinine concentration
UIC/Cr_Avg	average UIC/Cr of the two samples collected in the first and second trimester
UL	upper intake level
VIQ	verbal IQ
WHO	World Health Organization
WPPSI-III	Wechsler Preschool and Primary Scale of Intelligence, Third Edition
