# Supplementary material for: Urinary Iodine Concentration and Thyroid Hormone Metabolism in Pregnant Women and Neurodevelopment in Their Children: A Longitudinal Canadian Birth Cohort
Source: Nutrients. 2025 Feb 27;17(5):830. doi: 10.3390/nu17050830 (PMC11902198; doi:10.3390/nu17050830)
Supplement: Supplementary file 1 [file nutrients-17-00830-s001.zip › nutrients-3466564-supplementary.pdf]

<u>Supplementary Tables</u>

Supplementary Tables to “Maternal urinary iodine concentration and thyroid hormone metabolism in pregnancy and offspring neurodevelopment in a longitudinal Canadian birth cohort”

Authors: Sietske A. Berghuis, Meaghan Hall, John E. Krzeczkowski, Carly V. Goodman, Jonathan Chevrier, Pierre Ayotte, Bruce Lanphear and Christine Till

Table S1. Characteristics of the full sample (n=1831) and the sample with data on UIC/Cr, thyroid hormone data or neurodevelopmental outcome and complete data on primary covariates

Table S2. Spearman rank correlation test between thyroid parameters during the first trimester of pregnancy within the MIREC cohort

Table S3. Univariate linear and logistic regression analyses for maternal urinary iodine concentration divided by urinary creatinine concentration and thyroid hormone metabolism parameters during trimester 1 of pregnancy (excluding women using thyroid medication)

Table S4: Multivariable linear regression analyses for maternal urinary iodine concentration divided by urinary creatinine concentration and thyroid hormone metabolism during trimester 1 of pregnancy (excluding women using thyroid medication and with positive thyroid autoantibodies)

Table S5: Multivariable linear regression analyses for maternal urinary iodine concentration divided by urinary creatinine concentration in quartiles and thyroid hormone metabolism during trimester 1 of pregnancy (excluding women using thyroid medication)

Table S6. Univariate linear regression analyses for maternal urinary iodine concentration divided by urinary creatinine concentration during pregnancy (average T1 and T2) and neurodevelopmental outcomes in offspring (including woman using thyroid medication)

Table S7: Multivariable linear regression analyses for maternal urinary iodine concentration divided by urinary creatinine concentration during pregnancy in quartiles and neurodevelopmental outcomes in offspring

Table S8: Multivariable linear regression analyses for maternal urinary iodine concentration divided by urinary creatinine concentration during pregnancy in the first and second trimester and neurodevelopmental outcomes in offspring (n T1=796 T1 and n T2=783; including women using thyroid medication)

Table S9: Overview of studies on UIC/Cr and thyroid hormone parameters during the first trimester of pregnancy

Table S1. Characteristics of the full sample (n=1831) and the sample with data on UIC/Cr, thyroid hormone data or neurodevelopmental outcome and complete data on primary covariates

	Full sample singleton, live births (n=1831)	Thyroid hormone data and UIC/Cr T1[a] (n=1501)	Neurodevelopmental data and UIC/Cr_Avg [b] (n=760)
**Maternal characteristics**			
**Maternal age at pregnancy** (years; mean ± SD)	32.2 ± 5.1	32.3 ± 5.0	32.8 ± 4.7
**Race** (n;%)			
White	1557 (86%)[c]	1287 (86%)	690 (91%)
Non-white	259 (14%)[c]	214 (14%)	70 (9%)
**Level of education of mother at pregnancy** (n; %)			
College diploma or less	678 (37%) [c]	551 (37%)	241 (32%)
University undergraduate or graduate degree	1138 (63%) [c]	950 (63%)	519 (68%)
**Pre-pregnancy BMI** (kg/m^2; mean; SD)	24.9 ± 5.4 [d]	24.8 ± 5.4	25.0 ± 5.6
**Parity** (n; %)			
0	802 (44%)	661 (44%)	339 (45%)
1	742 (41%)	611 (41%)	310 (41%)
≥2	287 (16%)	229 (15%)	111 (15%)
**Alcohol use during pregnancy** [e](n; %)	290 (17%)	250 (18%)	134 (18%)
**Second-hand smoke during pregnancy** [f](n; %)	104 (5.7%)	83 (5.5%)	34 (4.5%)
**Use of prenatal vitamins** [g]	1590 (88%)	1314 (88%)	660 (87%)
**Child characteristics**			
Child male sex (n; %)	965 (53%) [h]	800 (53%)	364 (48%)

Abbreviations: UIC= urinary iodine concentration; Cr= creatinine; T1= trimester 1; SD= standard deviation; BMI= body mass index; CESD= Center for Epidemiological Studies Depression Scale; TgAb= thyroglobulin antibodies; TPOAb= thyroid peroxidase antibodies; HOME= Home Observation for Measurement of the Environment; WPPSI= Wechsler Preschool and Primary Scale of Intelligence; SRS= Social Responsiveness Scale;

[a] Data presented for subgroup with thyroid parameters is including pregnant women with UIC/Cr in the first trimester (T1) without using thyroid medication;

[b] Data presented for subgroup with neurodevelopment is including women with UIC/Cr in T1 and T2 and pregnant women using thyroid medication;

[c] 15 missing data; [d] 135 missing data; [e] women who reported ever drinking alcohol during pregnancy; 165, 127 and 19 missing data, respectively; [f] 18 and 2 missing for full sample and thyroid parameter subgroup, respectively; [g] 16 missing for full sample and 1 for other samples, respectively; [h] 7 missing data.

Table S2. Spearman rank correlation test between thyroid parameters during the first trimester of pregnancy within the MIREC cohort [a]

	**TSH** (µIU/mL)	**TT4** (ng/mL)	**FT4** (pg/mL)	**Tg** (ng/mL)	**TgAb** (IU/mL)
**TT4** (ng/mL) Rho	-0.074*				
p-value	0.006				
**FT4 (**pg/mL) Rho	-0.175*	0.317*			
p-value	<0.0001	<0.0001			
**Tg** (ng/mL) Rho	-0.049	-0.042	0.061*		
p-value	0.070	0.122	0.023		
**TgAb** (IU/mL) Rho	0.139*	-0.013	0.011	-0.179*	
p-value	<0.0001	0.627	0.684	<0.0001	
**TPOAb** (IU/mL) Rho	0.139*	-0.053*	-0.033	-0.109*	0.408*
p-value	<0.0001	0.049	0.217	<0.0001	<0.0001

[a] Data are presented for pregnant women with at least one thyroid parameter, data on all primary covariates and excluding women who reported taking thyroid hormone medication; * indicates *p*-value < 0.05; TSH: thyroid stimulating hormone; TT4: total T4; FT4: free T4; Tg: thyroglobulin; TgAb: Tg .antibodies; TPOAb: thyroid peroxidase antibodies.

Table S3. Univariate linear and logistic regression analyses for maternal urinary iodine concentration divided by urinary creatinine concentration and thyroid hormone metabolism parameters during trimester 1 of pregnancy (excluding women using thyroid medication)

	Crude Model [a]			
	n	**B**	**95 % CI**	***p***
**Log TSH** (uIU/mL)				
UIC/Cr T1 (μg/g×0.01)	1426	0.00	-0.02, 0.02	0.89
<150 versus 150-500 μg/g		-0.12	-0.23, -0.00	0.04*
≥500 versus 150-500 μg/g		-0.02	-0.14, 0.10	0.75
**Log fT4** (pg/mL)				
UIC/Cr T1 (μg/g×0.01)	1478	-0.00	-0.00, 0.00	0.83
<150 versus 150-500 μg/g		-0.01	-0.03, -.01	0.42
≥500 versus 150-500 μg/g		-0.02	-0.04, 0.01	0.21
**TT4** (ng/mL)				
UIC/Cr T1 (μg/g×0.01)	1499	-0.09	-0.53, 0.35	0.68
<150 versus 150-500 μg/g		2.25	-0.32, 4.82	0.09
≥500 versus 150-500 μg/g		1.32	-1.54, 4.19	0.37
**Log Tg** (ng/mL)				
UIC/Cr T1 (μg/g)	1485	-0.02	-0.04, 0.01	0.20
<150 versus 150-500 μg/g		0.18	0.04, 0.32	0.01*
≥500 versus 150-500 μg/g		0.03	-0.13, 0.18	0.73

Table S3. Univariate linear and logistic regression analyses for maternal urinary iodine concentration divided by urinary creatinine concentration and thyroid hormone metabolism parameters during trimester 1 of pregnancy (excluding women using thyroid medication)

	Crude Model [a]			
	n	**B**	**95 % CI**	***p***
**Logistic Regression**				
**TgAb ≥4.11 IU/mL** (versus <4.11)				
UIC/Cr T1 (µg/g×0.01)	1481			
<150 versus 150-500 µg/g		-0.08	-0.40, 0.25	0.64
≥500 versus 150-500 µg/g		-0.45	-0.85, -0.06	0.03*
**TPOAb ≥5.61 IU/mL** (versus <5.61)				
UIC/Cr T1 (µg/g×0.01)	1474			
<150 versus 150-500 µg/g		0.08	-0.29, 0.44	0.68
≥500 versus 150-500 µg/g		-0.59	-1.08, -0.10	0.02*

[a] Univariate analyses without any covariates in the model;
* indicates $p < 0.05$.

Table S4. Multivariable linear regression analyses for maternal urinary iodine concentration divided by urinary creatinine concentration and thyroid hormone metabolism during trimester 1 of pregnancy (excluding women using thyroid medication and women with positive thyroid autoantibodies)

	**Primary Model [a]**				**Secondary Model [b]**			
	n	**B**	**95 % CI**	***p***	***n***	**B**	**95 % CI**	***p***
**Log TSH** (uIU/mL)								
UIC/Cr T1 (μg/g)	1101				1012			
<150 versus 150-500 μg/g		-0.13	-0.26, -0.00	0.04*		-0.10	-0.23, 0.03	0.14
≥500 versus 150-500 μg/g		0.04	-0.09, 0.17	0.56		0.05	-0.09, 0.19	0.49
**Log fT4** (pg/mL)								
UIC/Cr T1 (μg/g)	1138				1044			
<150 versus 150-500 μg/g		-0.01	-0.03, 0.02	0.57		-0.01	-0.04, 0.02	0.53
≥500 versus 150-500 μg/g		-0.02	-0.05, 0.01	0.21		-0.01	-0.04, 0.02	0.40
**TT4** (ng/mL)								
UIC/Cr T1 (μg/g)	1149				1055			
<150 versus 150-500 μg/g		3.30	0.35, 6.25	0.03*		3.32	0.18, 6.46	0.04*
≥500 versus 150-500 μg/g		1.33	-1.80, 4.47	0.40		1.71	-1.54, 4.95	0.30
**Log Tg** (ng/mL)								
UIC/Cr T1 (μg/g)	1147				1053			
<150 versus 150-500 μg/g		0.16	0.06, 0.26	0.01*		0.17	0.07, 0.28	0.01*
≥500 versus 150-500 μg/g		-0.01	-0.12, 0.09	0.83		-0.01	-0.12, 0.10	0.91

[a] Primary Model: maternal age, race, pre-pregnancy body mass index, parity, child sex and age at sampling;
[b] Secondary Model: maternal age, race, maternal pre-pregnancy body mass index, parity, child sex, maternal alcohol use during pregnancy, second hand smoke during pregnancy and age at sampling;
* indicates *p*-value < 0.05.

Table S5. Multivariable linear regression analyses for maternal urinary iodine concentration divided by urinary creatinine concentration in quartiles and thyroid hormone metabolism during trimester 1 of pregnancy (excluding women using thyroid medication)

		Primary Model [a]			Secondary Model [b]		
		**B**	**95 % CI**	***p***	**B**	**95 % CI**	***p***
**Log TSH** (uIU/mL)							
UIC/Cr T1 (n=1426 and n=1305)	Q1 versus Q2+3	-0.14	-0.24, -0.03	0.02*	-0.12	-0.23, -0.00	0.05*
	Q4 versus Q2+3	-0.07	-0.18, 0.04	0.22	-0.07	-0.18, 0.04	0.22
**Log fT4** (pg/mL)							
UIC/Cr T1 (n=1478 and 1354)	Q1 versus Q2+3	-0.00	-0.03, 0.02	0.80	-0.01	-0.03, 0.02	0.61
	Q4 versus Q2+3	-0.01	-0.04, 0.01	0.21	-0.01	-0.03, 0.01	0.37
**TT4** (ng/mL)							
UIC/Cr T1 (n=1499 and 1372)	Q1 versus Q2+3	1.13	-1.37, 3.63	0.37	1.63	-1.00, 4.26	0.22
	Q4 versus Q2+3	-0.06	-2.55, 2.44	0.96	0.49	-2.09, 3.08	0.71
**Log Tg** (ng/mL)							
UIC/Cr T1 (n=1485 and 1358)	Q1 versus Q2+3	0.12	-0.02, 0.25	0.10	0.10	-0.05, 0.25	0.18
	Q4 versus Q2+3	-0.08	-0.22, 0.06	0.26	-0.09	-0.23, 0.06	0.23

Abbreviations: CI = confidence interval; Q1:<162.8 μg/g; Q2-3:162.8-423.7 μg/g; Q4≥423.7 μg/g;

[a] Primary Model: maternal age, race, pre-pregnancy body mass index, parity, child sex and age at sampling;

[b] Secondary Model: maternal age, race, maternal pre-pregnancy body mass index, parity, child sex, maternal alcohol use during pregnancy, second hand smoke during pregnancy and age at sampling;

* indicates *p*-value < 0.05.

Table S6. Univariate linear regression analyses for maternal urinary iodine concentration divided by urinary creatinine concentration during pregnancy (average T1 and T2) and neurodevelopmental outcomes in offspring (including woman using thyroid medication)

	Crude Model			
	n	B	95 % CI	*p*
**WPPSI- Full Scale IQ**				
UIC/Cr Average T1+T2 (μg/g×0.01)	503	-0.02	-0.36, 0.32	0.91
<150 versus 150-500 μg/g		-0.79	-4.55, 2.98	0.68
≥500 versus 150-500 μg/g		0.01	-3.13, 3.16	0.99
Log Tg [a]	406	0.10	-1.89, 2.09	0.92
**WPPSI- Verbal IQ**				
UIC/Cr Average T1+T2 (μg/g×0.01)	500	0.04	-0.30, 0.37	0.84
<150 versus 150-500 μg/g		-0.46	-4.19, 3.28	0.81
≥500 versus 150-500 μg/g		1.41	-1.69, 4.51	0.37
Log Tg [a]	404	-0.11	-2.05, 1.83	0.91
**WPPSI- Performance IQ**				
UIC/Cr Average T1+T2 (μg/g×0.01)	498	-0.07	-0.44, 0.31	0.72
<150 versus 150-500 μg/g		-0.70	-4.84, 3.44	0.74
≥500 versus 150-500 μg/g		-1.62	-5.09, 1.85	0.36
Log Tg [a]	401	0.29	-1.88, 2.46	0.79
**SRS- Total t-score**				
UIC/Cr Average T1+T2 (μg/g×0.01)	498	-0.04	-0.20, 0.11	0.59
<150 versus 150-500 μg/g		-0.71	-2.46, 1.05	0.43
≥500 versus 150-500 μg/g		-2.13	-3.05, -0.13	0.03*
Log Tg [a]	399	-0.07	-1.00, 0.87	0.89

Table S6. Univariate linear regression analyses for maternal urinary iodine concentration divided by urinary creatinine concentration during pregnancy (average T1 and T2) and neurodevelopmental outcomes in offspring (including woman using thyroid medication)

		Crude Model		
	n	**B**	**95 % CI**	***p***
**BASC- T-score Composite score Externalizing Problems**				
UIC/Cr Average T1+T2 (μg/g×0.01)	751	-0.09	-0.26, 0.08	0.30
<150 versus 150-500 μg/g		0.66	-1.27, 2.59	0.50
≥500 versus 150-500 μg/g		-1.37	-2.89, 0.16	0.08
Log Tg [a]	595	0.06	-0.88, 0.99	0.90
**BASC- T-score Composite score Internalizing Problems**				
UIC/Cr Average T1+T2 (μg/g×0.01)	744	0.07	-0.11, 0.25	0.45
<150 versus 150-500 μg/g		0.27	-1.80, 2.33	0.80
≥500 versus 150-500 μg/g		0.80	-0.83, 2.42	0.34
Log Tg [a]	588	-0.87	-1.86, 0.12	0.09

Abbreviations: CI = confidence interval;

[a] Excluding women with Anti-Tg ≥4.11 IU/mL or Anti-TPO >5.61 IU/mL, including women using thyroid medication.

Table S7. Multivariable linear regression analyses for maternal urinary iodine concentration divided by urinary creatinine concentration during pregnancy in quartiles and neurodevelopmental outcomes in offspring (for n with data on both T1 and T2, excluding women using thyroid medication)

		Primary Model [a]			Secondary Model [b]		
		B	95 % CI	*p*	B	95 % CI	*p*
**WPPSI- Full Scale IQ**							
UIC/Cr Average T1+T2 (n=503 and 455 for Models resp)	Q1 versus Q2+3	1.39	-1.34, 4.13	0.32	0.92	-1.86, 3.71	0.51
	Q4 versus Q2+3	0.48	-2.25, 3.21	0.73	0.20	-2.59, 2.99	0.89
**WPPSI- Verbal IQ**							
UIC/Cr Average T1+T2 (n=500 and 452 for Models resp)	Q1 versus Q2+3	1.00	-1.68, 3.69	0.46	0.54	-2.21, 3.29	0.70
	Q4 versus Q2+3	-0.05	-2.71, 2.62	0.97	-0.40	-3.15, 2.35	0.77
**WPPSI- Performance IQ**							
UIC/Cr Average T1+T2 (n=498 and 451 for Models resp)	Q1 versus Q2+3	1.25	-1.78, 4.27	0.42	1.08	-2.05, 4.20	0.50
	Q4 versus Q2+3	0.81	-2.21, 3.83	0.60	0.76	-2.39, 3.90	0.64
**SRS- Total t-score**							
UIC/Cr Average T1+T2 (n=498 and 451 for Models resp)	Q1 versus Q2+3	0.46	-0.85, 1.77	0.49	0.48	-0.86, 1.81	0.48
	Q4 versus Q2+3	-0.63	-1.94, 0.67	0.34	-0.42	-1.76, 0.93	0.54
**BASC- Composite score Externalizing Problems**							
UIC/Cr Average T1+T2 (n=751 and 455 for Models resp)	Q1 versus Q2+3	-0.23	-1.65, 1.20	0.75	-0.42	-2.16, 1.31	0.63
	Q4 versus Q2+3	-0.94	-2.34, 0.47	0.19	-1.30	-3.04, 0.44	0.14
**BASC- Composite score Internalizing Problems**							
UIC/Cr Average T1+T2 (n=744 and 453 for Models resp)	Q1 versus Q2+3	-1.18	-2.70, 0.34	0.13	-0.25	-2.02, 1.53	0.79
	Q4 versus Q2+3	-0.05	-1.56, 1.45	0.95	-0.24	-2.00, 1.57	0.81

Abbreviations: CI = confidence interval; Q1: <216.2 μg/g ; Q2-Q3: 216.2-446.6 μg/g; Q4: ≥446.6 μg/g;

[a] Primary Model: maternal age, race, pre-pregnancy body mass index, parity, maternal education level and study site;

[b] Secondary Model: maternal age, race, maternal pre-pregnancy body mass index, parity, maternal education level, child sex, maternal alcohol use during pregnancy, second hand smoke during pregnancy, depression CES-D 10 score mother, breast feeding, study site and HOME score.

Table S8. Multivariable linear regression analyses for maternal urinary iodine concentration divided by urinary creatinine concentration during pregnancy in the first and second trimester and neurodevelopmental outcomes in offspring (including women using thyroid medication)

	Primary Model [a]				Secondary Model [b]				Interaction term sex[c]
	n	B	95 % CI	*p*	*n*	B	95 % CI	*p*	*p*
**WPPSI- Full Scale IQ**									
UIC/Cr T1 (µg/g×0.01)	529	-0.22	-0.70, 0.26	0.37	469	-0.28	-0.75, 0.19	0.25	0.06
<150 versus 150-500 µg/g	529	0.92	-1.90, 3.74	0.52		0.39	-2.44, 3.22	0.79	0.05*
· boys as reference		3.99	-0.11, 8.10	0.06		2.98	-1.11, 7.07	0.15	·
· girls as reference		-1.80	-5.67, 2.07	0.36		-1.94	-5.84, 1.96	0.33	·
≥500 versus 150-500 µg/g		-0.19	-3.26, 2.88	0.90		-0.36	-3.40, 2.69	0.82	0.16
UIC/Cr T2 (µg/g×0.01)	518	0.02	-0.15, 0.20	0.78	469	0.04	-0.13, 0.22	0.64	0.26
<150 versus 150-500 µg/g		-1.03	-4.28, 2.22	0.53		-0.66	-4.04, 2.72	0.70	0.78
≥500 versus 150-500 µg/g		-0.68	-3.44, 2.08	0.63		-0.15	-2.95, 2.65	0.92	0.58
**WPPSI- Verbal IQ**									
UIC/Cr T1 (µg/g×0.01)	526	-0.21	-0.67, 0.26	0.39	466	-0.30	-0.76, 0.17	0.21	0.06
<150 versus 150-500 µg/g		0.53	-2.22, 3.28	0.70		0.47	-2.33, 3.28	0.74	0.07
· boys as reference		3.24	-0.78, 7.25	0.11		2.56	-1.51, 6.62	0.22	·
· girls as reference		-1.82	-5.57, 1.94	0.34		-1.37	-5.21, 2.47	0.48	·
≥500 versus 150-500 µg/g		-0.37	-3.34, 2.60	0.80		-0.82	-3.82, 2.17	0.59	0.14
UIC/Cr T2 (µg/g×0.01)	515	0.01	-0.16, 0.18	0.92	466	0.02	-0.15, 0.19	0.82	0.46
<150 versus 150-500 µg/g		-0.31	-3.49, 2.88	0.85		-0.04	-3.39, 3.30	0.98	0.18
≥500 versus 150-500 µg/g		-0.45	-3.13, 2.24	0.74		-0.34	-3.09, 2.41	0.81	0.68

Table S8. Multivariable linear regression analyses for maternal urinary iodine concentration divided by urinary creatinine concentration during pregnancy in the first and second trimester and neurodevelopmental outcomes in offspring (including women using thyroid medication)

	Primary Model [a]				Secondary Model [b]				Interaction term sex[c]
	n	B	95 % CI	*p*	*n*	B	95 % CI	*p*	*p*
**WPPSI- Performance IQ**									
UIC/Cr T1 (μg/g×0.01)	524	-0.09	-0.62, 0.44	0.74	465	-0.09	-0.62, 0.45	0.76	0.15
<150 versus 150-500 μg/g		0.82	-2.29, 3.93	0.61		0.03	-3.18, 3.23	0.99	0.13
≥500 versus 150-500 μg/g		-0.06	-3.46, 3.33	0.97		0.16	-3.30, 3.62	0.93	0.36
UIC/Cr T2 (μg/g×0.01)	513	0.04	-0.16, 0.23	0.72	465	0.05	-0.14, 0.25	0.60	0.27
<150 versus 150-500 μg/g		-1.66	-5.25, 1.94	0.37		-0.94	-4.74, 2.86	0.63	0.31
≥500 versus 150-500 μg/g		-0.75	-3.81, 2.31	0.63		0.12	-3.04, 3.28	0.94	0.52
**SRS- Total t-score**									
UIC/Cr T1 (μg/g×0.01)	523	-0.06	-0.28, 0.17	0.63	465	-0.01	-0.23, 0.22	0.94	0.68
<150 versus 150-500 μg/g		-0.33	-1.66, 1.00	0.63		-0.02	-1.38, 1.34	0.98	0.47
≥500 versus 150-500 μg/g		-0.89	-2.31, 0.54	0.22		-0.67	-2.12, 0.78	0.36	0.74
UIC/Cr T2 (μg/g×0.01)	514	-0.01	-0.10, 0.07	0.81	466	-0.01	-0.10, 0.07	0.75	0.45
<150 versus 150-500 μg/g		0.18	-1.38, 1.74	0.82		0.02	-1.60, 1.63	0.98	0.73
≥500 versus 150-500 μg/g		-0.46	-1.78, 0.87	0.50		-0.42	-1.76, 0.91	0.54	0.56

Table S8. Multivariable linear regression analyses for maternal urinary iodine concentration divided by urinary creatinine concentration during pregnancy in the first and second trimester and neurodevelopmental outcomes in offspring (including women using thyroid medication)

	Primary Model [a]				Secondary Model [b]				Interaction term sex[c]
	n	B	95 % CI	*p*	*n*	B	95 % CI	*p*	*p*
**BASC- Composite score Externalizing Problems**									
UIC/Cr T1 (μg/g×0.01)	786	-0.05	-0.29, 0.19	0.70	468	-0.09	-0.39, 0.20	0.54	0.73
<150 versus 150-500 μg/g		-0.23	-1.70, 1.24	0.76		0.55	-1.23, 2.32	0.54	0.11
≥500 versus 150-500 μg/g		-0.29	-1.80, 1.21	0.70		-0.41	-2.31, 1.49	0.67	0.77
UIC/Cr T2 (μg/g×0.01)	773	-0.04	-0.14, 0.05	0.39	470	-0.01	-0.12, 0.10	0.84	0.94
<150 versus 150-500 μg/g		0.92	-0.79, 2.62	0.29		0.68	-1.45, 2.82	0.53	0.28
>500 versus 150-500 μg/g		-0.02	-1.45, 1.42	0.98		-0.36	-2.12, 1.40	0.69	0.995
**BASC- Composite score Internalizing Problems**									
UIC/Cr T1 (μg/g×0.01)	779	0.20	-0.06, 0.46	0.13	466	0.01	-0.29, 0.32	0.93	0.89
<150 versus 150-500 μg/g		-0.64	-2.23. 0.95	0.43		0.64	-1.21, 2.48	0.50	0.47
≥500 versus 150-500 μg/g		0.61	-1.02, 2.23	0.47		-0.01	-1.99, 1.96	0.99	0.78
UIC/Cr T2 (μg/g×0.01)	766	0.02	-0.08, 0.12	0.74	468	0.05	-0.06, 0.16	0.35	0.07
<150 versus 150-500 μg/g		-0.91	-2.72, 0.90	0.32		-0.04	-2.20, 2.12	0.97	0.35
≥500 versus 150-500 μg/g		0.78	-0.74, 2.30	0.32		0.85	-0.94, 2.64	0.35	0.49

Abbreviations: CI = confidence interval;

[a] Primary Model: maternal age, race, pre-pregnancy body mass index, parity, maternal education level, child sex and study site;

[b] Secondary Model: maternal age, race, maternal pre-pregnancy body mass index, parity, maternal education level, child sex, maternal alcohol use during pregnancy, second hand smoke during pregnancy, depression CES-D 10 score mother, breast feeding, study site and HOME score;

[c] In primary model;

* indicates p-value < 0.05.

Table S9. Overview of studies on UIC/Cr and thyroid hormone parameters during the first trimester of pregnancy

Reference	Country	No. of participants with data in T1	Exclusion Criteria Study Group	Gestational age Median (wks)	UIC/Cr Median (in µg/g)	Thyroid hormone parameters								Comments
						TSH	fT4	TT4	fT3	TT3	Tg	Tg-Ab-pos	TPO-Ab-pos	
Luo et al 2021 [1]	China	167 (wk 8) 118 (wk 12)	Personal/family history of thyroid disease; palpable or visible goiter or nodules; abnormal thyroid function; TH-Ab-pos	8 (IQR:7-12)	111.49 (95%CI:38.63–489.46)	·	·	·	·	·	·	·	·	↑ Overt hypothyroidism (TSH>4.52 mU/L and fT4 <13.15 pmol/L)
					<38.63 vs. 38.63-489.46	·	·	·	·	·	·	↑	↑	
					>489.46 vs. 38.63-489.46	·	·	·	·	·	·	(↑)	N.S.	
Levie et al 2019 [2]	Sweden	2009	Thyroid medication; pre-existing thyroid disease; For analyses TH: TPO-Ab-pos	10 (95%: 4-16)	85 (95%CI:36–386)	↑	(↓)	↓	(↓)	(↓)	·	·	·	
					<150 vs. 150-249	·	·	·	·	·	·	N.S.	↑	
					250-499 vs 150-249	·	·	·	·	·	·	N.S.	N.S.	
					≥500 vs 150-249	·	·	·	·	·	·	N.S.	N.S.	
Bath et al 2017 [3]	United Kingdom	190	Thyroid medication; current smoking; taking selenium-containing supplement; TH-Ab-pos; UIC/Cr >700 µg/g (n=2)	12 (range: 9-14)	104 (IQR 66-172)	·	·	·	·	·	·	·	·	
					<150 vs ≥150	N.S.	·	·	·	·	(↑)	·	·	
					<100 vs 150-249	N.S.	·	·	·	·	N.S.	·	·	↑ Tg over 3 trimesters
					100-149 vs 150-249	N.S.	·	·	·	·	N.S.	·	·	↑ Tg over 3 trimesters
					>250 vs 150-249	N.S.	·	·	·	·	N.S.	·	·	
Moreno-Reyes et al 2013 [4]	Belgium	639	TPO-Ab-pos and Tg-Ab-pos; history of thyroid disease	9.9 (SD 2.8)	103 (IQR 71–172)	N.S.	↓ (T1+3)	·	↓ (T1+3)	·	↓	·	·	
					≥150 vs < 50	N.S.	·	·	·	·	↓ (T1&3)	·	·	
					<100 vs 100-149, 150-249 and ≥ 250	N.S.	·	·	·	·	(↑)	·	·	
Mullan et al 2022 [5]	Ireland	203	Thyroid disease; thyroid medication; UIC >500 µg/L (n=2)	<14	116	·	·	·	·	·	↓	·	·	
Zhang et al 2016 [6]	China	133	Abnormal TSH levels; TPO-Ab-pos and Tg-AB-pos; personal/family history of thyroid disease; history chronic disease; active/passive smoking, medication that may impact thyroid function	8	125.52 (90.83, 201.95)	·	·	·	·	·	N.S.	·	·	

Table S9. (*continued*) Overview of studies on UIC/Cr and thyroid hormone parameters during the first trimester of pregnancy

Reference	Country	No. of participants with TH data in T1	Exclusion Criteria Study Group	Gestational age Median (wks)	UIC/Cr Median (in μg/g)	Thyroid hormone parameters								Comments
						TSH	fT4	TT4	fT3	TT3	Tg	Tg-Ab-pos	TPO-Ab-pos	
Derakhshan et al 2018 [7]	United Kingdom	2362	Pre-existing thyroid disease; fertility treatment; twin pregnancies	10.9 (3.1)	117 (IQR 80 to 190)	N.S.	N.S.	·	·	·	·	·	(↑)	↑ TPO-Ab
Zhang et al 2019 [8]	China	79	Personal/family history of thyroid disease; TSH ≥ 2.5 mIU/L; TPO-Ab-pos and Tg-Ab-pos; goiter; thyroid-related medication; gynecologic condition; multivariable pregnancy; UIC ≥ 150 μg/L	12	188.26 (144.81~256.74)	·	·	·	·(see comment)	·	·(see comment)	·	·	No report on UICr; Unadusted UIC: ↓Tg 250–499 μg/L compared to 150–249 μg/L
Konrade et al 2015 [9]	Latvia	155	Known pre-existing thyroid disease	n.r. (T1)	56.0 (IQR 36.4–100.6); <150	·	·	·	·	·	·	·	N.S.	
Fuse et al 2011 [10]	Japan	243	History of thyroid disease	9.3 (SD 1.8)	185.4 (IQR: 113.0 –350.0)	N.S.	N.S.	·	·	·	·	N.S.(see comment)	N.S. (see comment)	Data presented for THab positive
Orito et al. 2009 [11]	Japan	514	Thyroid disorder/complication; TPO-Ab-pos and pos TSH binding inhibitor immunoglobulin; twin pregnancies	Mean: 10.5 (SD 4.8)	259.5 (16.0–5353)	↑	↓	·	↓	·	·	·	·	
Current study	Canada	1501	Use of thyroid medication	Mean: 11.6 (SD 1.5)	266.5 (162.8-423.7)	·	·	·	·	·	·	·	·	
					<150 vs 150-500	N.S.	N.S.	(↑)	·	·	↑	N.S.	N.S.	
					≥500 vs 150-500	N.S.	N.S.	N.S.	·	·	N.S.	N.S. (see comment)	N.S. (see comment)	If TH-Ab-pos excluded: ↓ anti-TPO and ↓ anti-Tg

Data are reported for studies reporting on UIC/Cr and TH parameters measured during the first trimester of pregnancy; UIC/Cr: urinary iodine concentration divided by urinary creatinine concentration;
↑ indicates positive association between continuous UIC/Cr or 1st mentioned UIC/Cr category (compared to 2nd category) with TH parameter (P<.05); (↑) indicates marginally significant positive association (p<.10);
↓ indicates negative association between continuous UIC/Cr or 1st mentioned UIC/Cr category (compared to 2nd category) with TH parameter; (↓) indicates marginally significant negative association (p<.10);
'.' indicates data not reported; 'N.S.': not significant;

References Table S9

[1] Luo, J.; Li, C.; Zhang, X.; Shan, Z.; Teng, W. Reference Intervals of the Ratio of Urine Iodine to Creatinine in Pregnant Women in an Iodine-Replete Area of China. Biol. Trace Elem. Res. **2021**, *199*, 62–69.

[2] Levie, D.; Derakhshan, A.; Shu, H.; Broeren, M.A.; De Poortere, R.A.; Peeters, R.P.; Bornehag, C.; Demeneix, B.; Korevaar, T.I. The Association of Maternal Iodine Status in Early Pregnancy with Thyroid Function in the Swedish Environmental Longitudinal, Mother and Child, Asthma and Allergy Study. Thyroid **2019**, *29*, 1660–1668.

[3] Bath, S.C.; Pop, V.J.; Furmidge-Owen, V.L.; Broeren, M.A.; Rayman, M.P. Thyroglobulin as a Functional Biomarker of Iodine Status in a Cohort Study of Pregnant Women in the United Kingdom. Thyroid **2017**, *27*, 426–433.

[4] Moreno-Reyes, R.; Glinoer, D.; Van Oyen, H.; Vandevijvere, S. High Prevalence of Thyroid Disorders in Pregnant Women in a Mildly Iodine-Deficient Country: A Population-Based Study. The Journal of Clinical Endocrinology & Metabolism **2013**, *98*, 3694–3701.

[5] Mullan, K.; McMullan, P.; Kayes, L.; McCance, D.; Hunter, A.; Woodside, J.V. Thyroglobulin Levels among Iodine Deficient Pregnant Women Living in Northern Ireland. Eur. J. Clin. Nutr. **2022**, *76*, 1542–1547.

[6] Zhang, X.; Li, C.; Mao, J.; Wang, W.; Xie, X.; Peng, S.; Wang, Z.; Han, C.; Zhang, X.; Wang, D. Gestation-specific Changes in Maternal Thyroglobulin during Pregnancy and Lactation in an Iodine-sufficient Region in China: A Longitudinal Study. Clin. Endocrinol. (Oxf) **2017**, *86*, 229–235.

[7] Derakhshan, A.; Korevaar, T.I.; Taylor, P.N.; Levie, D.; Guxens, M.; Jaddoe, V.W.; Nelson, S.M.; Tiemeier, H.; Peeters, R.P. The Association of Maternal Thyroid Autoimmunity during Pregnancy with Child IQ. The Journal of Clinical Endocrinology & Metabolism **2018**, *103*, 3729–3736.

[8] Zhang, H.; Wu, M.; Yang, L.; Wu, J.; Hu, Y.; Han, J.; Gu, Y.; Li, X.; Wang, H.; Ma, L. Evaluation of Median Urinary Iodine Concentration Cut-Off for Defining Iodine Deficiency in Pregnant Women After a Long Term USI in China. Nutrition & metabolism **2019**, *16*, 1–9.

[9] Konrade, I.; Kalere, I.; Strele, I.; Makrecka-Kuka, M.; Jekabsone, A.; Tetere, E.; Veisa, V.; Gavars, D.; Rezeberga, D.; Pīrāgs, V. Iodine Deficiency during Pregnancy: A National Cross-Sectional Survey in Latvia. Public Health Nutr. **2015**, *18*, 2990–2997.

[10] Fuse, Y.; Ohashi, T.; Yamaguchi, S.; Yamaguchi, M.; Shishiba, Y.; Irie, M. Iodine Status of Pregnant and Postpartum Japanese Women: Effect of Iodine Intake on Maternal and Neonatal Thyroid Function in an Iodine-Sufficient Area. The Journal of Clinical Endocrinology & Metabolism **2011**, *96*, 3846–3854.

[11] Orito, Y.; Oku, H.; Kubota, S.; Amino, N.; Shimogaki, K.; Hata, M.; Manki, K.; Tanaka, Y.; Sugino, S.; Ueta, M. Thyroid Function in Early Pregnancy in Japanese Healthy Women: Relation to Urinary Iodine Excretion, Emesis, and Fetal and Child Development. The Journal of Clinical Endocrinology & Metabolism **2009**, *94*, 1683–1688.
